# Efficacy of a short-term webcam-based telemedicine treatment of internet use disorders (OMPRIS): a multicentre, prospective, single-blind, randomised, clinical trial

**DOI:** 10.1016/j.eclinm.2023.102216

**Published:** 2023-09-14

**Authors:** Jan Dieris-Hirche, Laura Bottel, Jale Basten, Magdalena Pape, Nina Timmesfeld, Bert Theodor te Wildt, Birte Linny Geisler, Klaus Wölfling, Peter Henningsen, Manfred Beutel, Anja Neumann, Anja Niemann, Rainer Beckers, Stephan Herpertz, OMPRIS Study Group, Annika Best, Raffaela Böswald, Lorraine Cornelsen, Michael Dreier, Sofie Groen, Alicia Hemmersbach, Vivienne Hillerich, Ina Krahn, Dennis Lowin, Alicia Menze, Silke Neusser, Nehle Penning, Heribert Sattel, Christian Suelmann, Marianne Tokic, Julia Weretecki

**Affiliations:** a Department of Psychosomatic Medicine and Psychotherapy, LWL-University Hospital, Ruhr University Bochum, Alexandrinenstraße 1-3, 44791, Bochum, Germany; b Department of Medical Informatics, Biometry and Epidemiology, Ruhr University Bochum, Universitätsstraße 105, 44789, Bochum, Germany; c Psychosomatic Hospital Diessen Monastery, Klosterhof 20, 86911, Diessen, Germany; d Department of Psychosomatic Medicine and Psychotherapy, University Hospital Rechts der Isar, Technical University Munich, Ismaninger Str. 22, 81675 Munich, Germany; e Outpatient Clinic for Behavioral Addictions, Department of Psychosomatic Medicine and Psychotherapy, University Medical Center of the Johannes Gutenberg-University Mainz, Untere Zahlbacher Str. 8, 55131 Mainz, Germany; f Institute for Health Care Management and Research, University Duisburg-Essen, Thea-Leymann-Str. 9, 45127, Essen, Germany; g Competence Centre of Healthcare Telematics, Haus Harkorten 8, 58135, Hagen, Germany

**Keywords:** Internet use disorder, Online therapy, Gaming disorder, Telemedicine, Treatment, Internet addiction

## Abstract

**Background:**

Evidence-based treatments for internet use disorders (IUDs) are limited, and online therapy approaches are poorly studied. We investigated the efficacy of a manualised therapist-guided online intervention (OMPRIS) to reduce IUD symptoms and improve psychological well-being.

**Methods:**

In this multicentre, two-arm, single-blinded trial, individuals ≥16 years suffering from IUD symptoms were recruited in Germany from August 16, 2020, to March 11, 2022, through media advertisements and healthcare providers. Participants were randomly allocated by sequential balancing randomisation (1:1) to either the manualised webcam-based OMPRIS intervention or a waitlist control (WLC) group. OMPRIS provided strategies from motivational interviewing, behavioural therapy, and social counselling. The primary outcome at the end of treatment was a reduction in IUD symptoms as measured by the Assessment of Internet and Computer Scale (AICA-S). Follow-up assessments were conducted at 6 weeks and 6 months. Analyses were performed in the intention-to-treat population. This trial was registered (German Clinical Trial Register, DRKS00019925) and has been completed.

**Findings:**

A total of 180 individuals were randomly assigned to the OMPRIS intervention (n = 89) or WLC (n = 91) arm. After treatment, 81 (91.0%) participants in the OMPRIS intervention group and 88 (96.7%) in the WLC group completed the outcome assessment. The ANCOVA model showed that OMPRIS participants had a significantly greater reduction in AICA-S scores from baseline (mean score 12.1 [SD 4.6]) to post-treatment (6.8 [5.2]) than those in the WLC group (from 12.6 [5.1] to 11.0 [5.4]; estimated mean difference −3.9; [95% CI −5.2 to −2.6]; p < 0.0001; d = 0.92). No adverse events were reported to the trial team.

**Interpretation:**

Webcam-based OMPRIS therapy was effective and superior to waiting list conditions in reducing IUD symptoms. Webcam-based, specialised online therapy thus increases IUD treatment options.

**Funding:**

German Innovation Fund of 10.13039/501100014840Germany’s Federal Joint Committee (G-BA), grant number 01VSF18043.

**Research in context**

**Evidence before this study**

A systematic literature search of PubMed and Web of Science identified 15 appropriate articles on the efficacy of several digital health interventions for Internet Use Disorders (IUDs), including 2 systematic reviews, 2 ongoing RCTs protocols, 2 RCTs, and several non-RCT studies focusing on different digital health approaches. Regarding online-based psychotherapy for IUDs, only 2 RCTs (conducted on college students and patients with problematic pornographic use) and a single-arm study from our own research group reported positive effects of an online/webcam-based intervention. In summary, internet-based interventions for IUDs have not yet been adequately studied, and further extensive research is needed to evaluate their efficacy.

**Added value of this study**

This is the first randomized, multicentre, single-blind, superiority trial to demonstrate that webcam-based telemedicine is effective in treating internet use disorders and gaming disorders. Our findings show that short-term, manualised, webcam-based online therapy reduces symptoms of internet use disorders, depression, and anxiety, while increasing motivation for change and self-efficacy. We also assessed symptom progression up to 6 months after intervention, making it possible to compare with previous in-person psychotherapy results.

**Implications of all the available evidence**

Our study significantly increases the evidence for telemedicine IUD treatment, especially using “home-based therapy” for people with IUD and gaming disorders. It is a valuable option for those with motivational issues, feelings of shame, or resistance to clinic treatment, and those with limited access to outpatient therapy due to long waiting lists or distance. Further research should explore factors affecting suitability for IUD subgroups.

## Introduction

The widespread use of the internet has brought numerous benefits, but it has also led to the emergence of a new type of addiction: internet use disorder (IUD).^1^ IUD is a condition characterised by excessive and uncontrolled use of the internet that can negatively impact an individual's personal, social, or occupational life. It includes both internet gaming disorder (IGD) and non-gaming activities, such as online shopping, online streaming, pornography and social media use.^2^ Researchers have started using the term “IUD” instead of “internet addiction” in order to align with the inclusion of gaming disorder as the most frequent subtype of IUD in the current International Classification of Diseases (ICD-11).^3^ IUD is considered a behavioural addiction due to its focus on online behaviours.^1^ The prevalence of IUDs has increased significantly in recent years, with rates ranging from 2.6% in Western and Northern Europe to 10.9% in the Middle East.^4^ A recent systematic review reports 7.02% as a global prevalence rate for IUD.^5^ This trend is likely to have been exacerbated by the COVID-19 pandemic, which led to increased internet usage and gaming behaviour among college students and adolescents.^6^

People with IUD may have difficulty controlling the onset, intensity, and duration of their internet use and may prioritise it over other activities and interests.^7^ IUD can lead to physical, social, and mental burden and is often accompanied by other mental disorders, such as depression, anxiety, attention deficit hyperactivity disorder, substance use disorders, and impulse control disorders.^8^

There are few treatment options currently available for IUD. Meta-analyses found that cognitive behavioural therapy (CBT) is effective in reducing IUD symptoms, depression, and anxiety.^9^, ^10^, ^11^, ^12^ Thus, there is substantial evidence for the positive therapeutic effects of analogue face-2 face CBT. Additional treatment approaches for IUD, such as medication, family therapy, and specialized treatment interventions, have been studied in initial research. However, due to limitations in the methodology of these studies, it is difficult to draw valid conclusions about the effectiveness of these interventions.^13^ Medication trials examined drugs typically applied to treat depression (bupropion, escitalopram) or ADHD (methylphenidate, atomoxetine). Some drug trials used a simple pretest-posttest design. Other studies presented head-to-head comparisons of two different drugs. However, neither study had a placebo control group. Only two medication (bupropion and escitalopram) studies used randomised designs with control groups.^13^ So far there are only a few studies on alternative psychotherapeutic interventions (family therapy, brief voluntary abstinence, transcranial direct stimulation, eclectic treatment approach, self-discovery camp) that cannot be classified as CBT. All of these studies were conducted in a pretest-posttest design. Some had very small case numbers or low IUD symptoms (non-clinical). Only two studies used non-randomised (7-days residential camps) or a randomised control-group design (writing course).^13^

Motivational interviewing (MI) is another treatment approach that has been discussed as a potential approach for IUD.^14^ With reference to the transtheoretical model of behavioural change (TTM) according to Prochaska and Di Clemente, MI is considered a suitable therapeutic approach in the early motivational phases (pre-contemplation), i.e. when the intention to change arises.^15^ The evidence for MI as a therapeutic approach in addiction therapy is very strong. MI has been widely used in the treatment of substance-related addictions and pathological gambling and may be effective for IUD as well.^16^^,^^17^

The use of telemedicine for treating individuals with substance abuse and behavioural addictions, such as alcohol abuse, smoking, and pathological gambling, has been on the rise in recent years. Studies have been conducted on the use of telemedicine interventions for gambling, using a variety of methods including videoconferencing, telephone, email, and chat.^18^^,^^19^ These studies generally show that telemedicine interventions are effective in reducing problem gambling scores and behaviours. However, it is noted that there is a lack of comparison with in-person interventions and diversity in samples, which requires further investigation.^20^

Currently, there have only been a very limited number of studies examining the use of eHealth interventions specifically for IUD patients. Two systematic reviews from 2016 to 2022 found only a few (pilot) studies, respectively, that examined different eHealth approaches (e.g. virtual reality exposure, cognitive bias modification, web-based self-help) for IUD patients.^18^^,^^21^ A Chinese pilot study recruited 65 university students who scored high for problematic internet use, and assigned them to four different experimental groups. The study found that there were significant changes in the follow-up measurements, however, it did not find any differences between the four groups that received different interventions.^22^ Another study published in 2021 was conducted on almost exclusively men with problematic pornography use and showed a significant treatment effect of a web-based self-help tool including CBT and MI interventions.^23^ Furthermore, our research unit was able to show in a pilot study that IUD patients can generally be reached for counselling via the internet.^24^

To our knowledge, there has not yet been a randomised controlled trial investigating the efficacy of a webcam-based specialised telemedicine treatment for IUD patients. Furthermore, a recent review published in 2022 by leading experts in the field of IUD research highlights the need for further research to address key gaps in knowledge about effective preventive and therapeutic interventions.^25^ As a result, this first multicentre, controlled, and randomised trial aims to examine the efficacy of a new online-based motivational intervention program for internet use disorders called OMPRIS. We hypothesise that the OMPRIS intervention will be effective in reducing IUD symptoms (hypothesis 1) as well as other psychological symptoms (hypothesis 2), and will increase motivation to change behaviour in terms of media use (hypothesis 3).

## Methods

### Study design

A multicentre, prospective, single-blinded, randomised controlled trial was conducted with therapists from four IUD specialized medical centres in Germany. Recruitment, diagnostics and intervention were carried out completely online. Allocation to the centres was random. The study was approved by the ethics committee for the Faculty of Medicine, Ruhr University Bochum (approval no. 19-6779) and by the ethics committees of the participating study centres located at university hospitals in Munich and Mainz. For more details see the study protocol.^26^

### Participants

Potential participants were recruited nationwide in Germany through advertisements in media, newspapers, podcasts, radio reports, and online search engines. Furthermore, potential participants could also be referred by experts, e.g. psychotherapists or addiction counselling centres. Those interested in participating were referred to the OMPRIS study website (www.onlinesucht-hilfe.com) with information about the study and treatment. Finally, individuals could start the application process by completing a quick self-test including the Assessment of Internet and Computer Game Addiction (AICA-S).^27^ In this project, both more severely affected people and those still in an early phase of IUD were addressed. Individuals who either screened positive or had high levels of subjective suffering were offered an initial webcam consultation with a trained clinical psychologist with a Master's degree and extensive experience in standardised psychiatric diagnostical assessments including the Mini International Neuropsychiatric Interview (MINI 7.0) for psychiatric comorbidity and the Assessment of Internet and Computer Game Addiction Structured Clinical Interview for diagnostic on IUD (AICA-SKI:IBS).^28^^,^^29^

Inclusion criteria were defined as follows: being at least 16 years old, presenting either hazardous/pathological use of internet applications according to the DSM-5 criteria and the ICD-11 criteria or at least subjective suffering regarding the own internet use, fulfilling the technical and language requirements, and informed consent to reverse the pseudonymisation in case of emergency (i.e. serious suicidal intents or plans). Exclusion criteria were defined as: presence of acute psychotic disorders, acute risk of suicide, severe intellectual impairments, presence of substance abuse, somatic diseases with endocrinal medication causing impulsive behaviour, undergoing current treatment focusing primarily on IUD. Participants were also excluded if they consumed illegal content (added later as an exclusion criterion; not mentioned in the study protocol as an exclusion criterion). For further inclusion details see the study protocol.^26^ Participants were informed at the beginning of the first webcam session and encouraged to ask questions. The consent forms could be downloaded from the study website at any time. All participants provided informed consent. For participants under 18 years of age, parental consent was obtained via webcam. Participants were offered vouchers completing online questionnaires on time but did not receive further financial incentives. All participants received a short, automatically generated feedback report by email after their last session. This included confirmation of participation, an IUD diagnosis, and (if relevant) a personalized recommendation for further treatment.

### Randomisation and masking

The participants were automatically assigned in a 1:1 ratio either to the OMRPIS intervention or the waitlist control group (WLC). Sequential balancing randomisation according to Borm et al.^30^ was used considering 4 relevant factors^24^: 1) gender (women, men, diverse); 2) severity of IUD symptoms (AICA-S score <7, 7–13; >13); 3) age (16–17, 18–30, >30 years); and 4) the type of IUD (video gaming, pornography/cybersex, all other genres). For further details see the study protocol.^26^ After randomisation, the OMPRIS platform automatically arranged the first online appointments for diagnostics. Different therapists conducted diagnostic and therapeutic interventions. Since all the participants had received treatment, therapists were unknown to allocate to the OMPRIS group or WLC group. Assessor blinding was achieved through software-based outcome measurement of all primary and secondary outcomes, which automatically stored patient self-reported data. The data review was maintained as blind before performing analyses. Data analyses were conducted with blinded group assignment.

### Procedures

A multicentre study was conducted with therapists from four university medical centres in Germany specializing in Internet use disorders (IUD): (1) LWL-University Hospital of the Ruhr University Bochum, Department of Psychosomatic Medicine and Psychotherapy; (2) Psychosomatic Hospital Diessen Monastery/Ruhr University Bochum; (3) Department of Psychosomatic Medicine and Psychotherapy, University Hospital Rechts der Isar, Technical University Munich; and (4) Outpatient Clinic for Behavioral Addictions, Department of Psychosomatic Medicine and Psychotherapy, University Medical Center of the Johannes Gutenberg-University Mainz. Recruitment, diagnosis, and therapy were conducted online. Therefore, OMPRIS provides location-independent therapy for all statutory health insurance-covered patients. An OMPRIS software environment was implemented using a protected database in Germany where study data were documented, monitored, and stored. Sensitive participant data was stored separately from research data in a second database. An initial pilot study was conducted between 2016 and 2018 to test trial procedures, technical issues, and recruitment strategies.^24^

The OMPRIS intervention was manualised and provided therapeutic guidance regarding the adaptations for the webcam-based application.^31^ The manual incorporated techniques from motivational interviewing (MI) with therapy tools from cognitive behavioural therapy (CBT) as well as acceptance and commitment therapy (ACT), which have been shown as sufficient to improve health behaviours in behavioural addictions including IUD.^11^ Furthermore, social counselling was offered for social problems. With reference to the transtheoretical model of behavioural change, MI is considered a suitable therapeutic approach in the early motivational phases (pre-contemplation), i.e. when the intention to change arises.^15^ Due to the good evidence and already existing CBT manuals, central therapeutic interventions were integrated into the OMPRIS manual (e.g. development of an individual disorder model, vicious circles, alternative behaviours, cognitive patterns and bias, usage protocols with SORC scheme). From the ACT approach, interventions focusing on the acceptance of certain emotional states (e.g. boredom, listlessness, silence, frustration) and the development of life goals and life values as motivational promoters were included in the OMPRIS manual. Furthermore, clinical experience shows chronic IUD patients often develop severe social problems (e.g. debts, loss of social and financial support, littering in the apartment). Therefore, basic social counselling was included in OMPRIS. For detailed information on the development of the OMPRIS manual, the therapeutic approaches used and the helpful therapeutic attitude, see the associated publication of our research group.^31^ In total, participants underwent 4 weeks of telemedicine intervention with at least 2 psychotherapeutic sessions per week and 1 or 2 social counselling sessions (approximately 60 min per session). An overview of the treatment phases and therapeutic approaches can be found in the supplements (eAppendix 1, Table S1). The WLC group received no intervention. However, they were offered participation in the OMPRIS intervention after the 4 weeks waiting period. In the middle of the waiting period, the WLC group received an email with brief information about the upcoming start of their treatment. A total of 7 trained psychotherapists (6 women, 1 man) conducted the OMPRIS intervention. Diagnostics were performed by a separate psychologist. All therapists had at least a Master's degree and were clinically experienced psychologists or experts in related disciplines. They also had experience treating patients with IUDs. Social counselling sessions were conducted by a female social worker. All therapists completed a one-day training session on the OMPRIS intervention provided by the treatment developers as well as several days of motivational interviewing training. Furthermore, all therapists received the treatment manual including all worksheets.

Treatment fidelity was assessed using therapist feedback after each session with a classification of the most significant themes and interventions. Core sessions were defined in the manual, which was mandatory.^31^ In addition, individual topic content was provided, which was used when appropriate (e.g. procrastination). The therapists received weekly supervisions from experienced psychotherapists.

### Outcomes

The primary outcome chosen was IUD symptom severity, which was measured using the Assessment of Internet and Computer Game Addiction Scale (AICA-S).^27^ The scale consists of 14 items (five-point Likert scale) that are related to the DSM-5 criteria for substance-use disorders and gambling disorder and includes craving, loss of control, tolerance, unsuccessful attempts to spend less time online, and withdrawal. Furthermore, it assesses negative consequences in school, work, health, and with social partners. Moreover, time spent online, the preferred online activities, and the preferred type of problematic internet use are requested. A score between 7.0 and 13.0 points is classified as moderately addictive internet use, and a score above 13.5 points is considered addictive internet use. The reliability of the AICA-S (internal consistency of α = 0.89) and validity are determined in clinical and epidemiological surveys.^32^ Secondary outcomes included depression symptoms (PHQ-9^33^), anxiety symptoms (GAD-7^34^), motivational stage of readiness and treatment eagerness (iSOCRATES^35^), global life satisfaction (L-1^36^), and general self-efficacy (GSE^37^). Depression and anxiety were assessed because depression and social anxiety disorders are among the most common psychiatric comorbidities in IUD.^8^ The motivation for change was investigated because this was also a central goal of the OMPRIS intervention. Life satisfaction and self-efficacy were surveyed to assess possible effects on (social) life and self-confidence. In addition, socio-demographic data as well as an internet usage history with usage times and frequencies, types of usage, and a global measure of pathological internet use (CIUS^38^) were examined. Finally, the Big Five personality traits (BFI-10^39^) were surveyed exploratively, since IUD patients often show a specific personality pattern.^40^ Detailed information on quality criteria and measurement times of secondary outcomes can be found in the study protocol.^26^ A standard operating procedure has been developed for the case of severe adverse events (e.g. suicide attempts). Suicidality was checked and documented in each session.

### Statistics

The sample size was calculated using a two-sided two-sample t-test at a significance level of 5% and to achieve a power of 80% to detect a difference of 2 points in the AICA-S assuming a standard deviation of 3.92 (effect size = 0.51 corresponding to 43% of the observed effect in the STICA study^10^). This resulted in a required sample size of 62 patients per group. Assuming a drop-out rate of 30%, 81 patients per group should be included in the study. For further details see the statistical analysis plan (online supplement) and the published study protocol.^26^ The primary and secondary outcome scores were measured at baseline (T0), mid-treatment (approximately 2 weeks after treatment start; T1), post-treatment (approximately 4 weeks after treatment start; T2), 6 weeks after intervention (6-week follow-up; T3) and 6 months after randomisation (6-months follow-up; T4). They were descriptively analysed by treatment arm across measurement points. The analysis of the primary outcome score AICA-S applied the intention-to-treat (ITT) principle and analysed all randomised participants. The primary endpoint for the RCT analysis was T2 (posttreatment), the primary outcome was the AICA-S. The follow-up measurements after 6 weeks and 6 months in the intervention group only served to record the course of the symptom burden. The secondary outcomes were evaluated in modified ITT populations. These included all randomised participants whose respective secondary outcomes at T0 and at T2 were measured. All analyses were performed by an analysis of covariance (ANCOVA), i.e. a linear regression analysis in which the post-treatment measurement (T2) of the respective score was used as dependent variable and the baseline pre-treatment measurement (T0) was used as a covariate. Further (co-)variates in the model were the intervention group, type of IUD, age, gender, and a binary indicator of whether any psychiatric comorbidity is currently present. The reason for the selection of the covariates was that gender as well as the complexity of pathology (comorbidity) may affect the type and extent of internet use.^41^ These factors were therefore also taken into account in the randomisation process. The type of IUD was grouped into (1) online streaming, (2) online gaming, (3) online pornography, (4) social networking sites and chatting, and (5) other genres (online shopping, information research, online gambling, and writing emails). A current psychiatric comorbidity was present if any comorbidities were reported in the MINI 7.0.2 collected at baseline. A detailed list of comorbid mental disorders can be found in the online supplement (eAppendix 2, Table S2). If the post-treatment (T2) measurement of the AICA-S primary outcome score was not available, it was replaced by the AICA-S at time T1, if available. If the AICA-S was not available at time T1 and T2, multiple imputation was performed using regression models. For this purpose, a linear regression model was fitted with covariates AICA-S at T0, age, gender, and type of IUD to make predictions for the post-treatment (T2) measurement of the AICA-S. The missing data were replaced by the corresponding predictions. Furthermore, a per-protocol analysis and sensitivity analyses for the primary outcome were conducted. All analyses are repeated for the per-protocol population consisting of all participants who participated in at least two online sessions (applied only to the OMPRIS intervention group) and completed the T2 survey 28 days after T2 release at the latest. For details on further analyses see the supplement (eAppendix 3–4). Cohens’ *d* was estimated from the regression model and used as an effect size indicator. By conducting a moderator analysis, we investigated possible predictors (e.g. person-related characteristics) for successful participation in the OMPRIS intervention. For this purpose, models with interactions between the intervention and the severity of IUD at baseline (AICA-S at T0), age, gender, and type of IUD were calculated. All covariates included in the regression model and moderator analysis had been collected completely from all participants. All statistical analyses were performed in R, version 4.2.1, and used a two-sided significance level of 5%. An independent, external safety monitoring board was established, consisting of experts in the field of statistics and behavioural addictions. The study has been registered on the German Clinical Trials Register (DRKS00019925).

### Ethics

Ethics committee approvals were obtained from the following ethics committees: Ethics Committee of the Medical Faculty of the Ruhr University Bochum (reference number: 19-6779), Ethics Committee of the Technical University of Munich (reference number: 127/20 S-KH), Ethics Committee of the Rhineland-Palatinate State Medical Association (reference number: 2020-15239). All participants provided informed consent. For participants under 18 years of age, parental consent was obtained via webcam.

### Role of the funding source

The funder of the study had no role in study design; data collection, data analysis, data interpretation, writing of the manuscript, and the decision to submit it for publication.

## Results

**Fig. 1:**
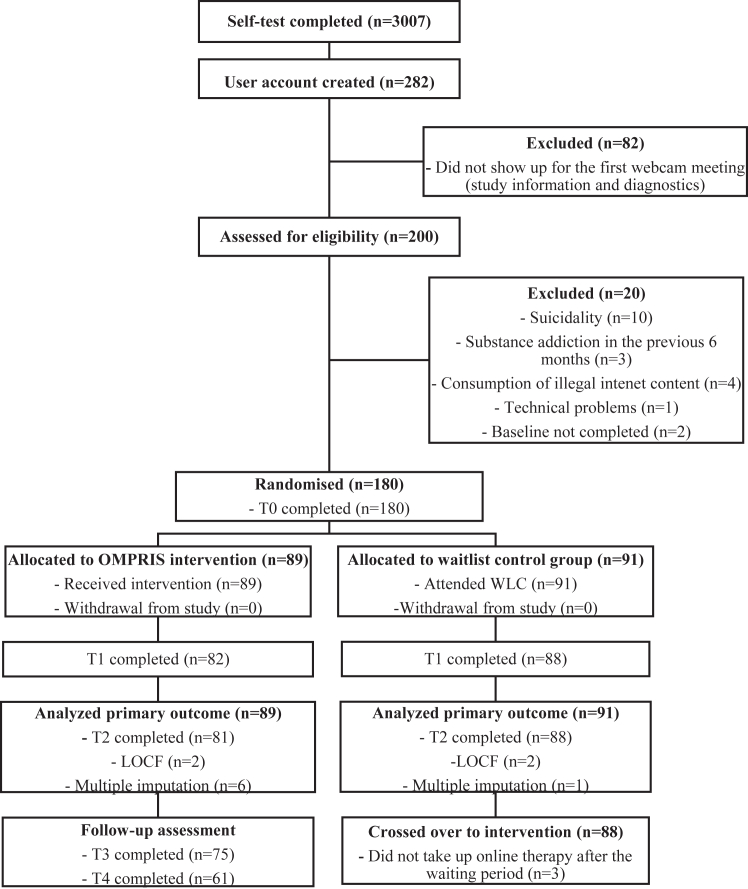
Trial profile. T0 indicates time factor, baseline; T1, time factor, mid-treatment (approximately 2 weeks after treatment start); T2, time factor, post-treatment (approximately 4 weeks after treatment start); T3, time factor, 6-week follow-up; and T4, time factor, 6-month follow-up; LOCF, last observation carried forward, i.e. if AICA-S at T2 is missing the primary outcome is the AICA-S at T1; WLC, waitlist control group.

The results are reported according to the Consolidated Standards of Reporting Trials (CONSORT) guidance.^42^ A total of 3007 participants completed the screening questionnaire via the OMPRIS platform between August 16, 2020, and March 11, 2022. Of these participants, 41.9% stated that they were sent by other people (e.g. parents, schools, medical experts), while 46.0% stated that they had found OMPRIS themselves through internet searches or press articles. A detailed description of this population has recently been published elsewhere by our research group.^43^ Of 200 participants assessed for eligibility, 180 participants were randomly assigned to the OMPRIS intervention or WLC group (Fig. 1). Socio-demographics, morbidity-related characteristics, and extent and type of addictive behaviour characteristics at baseline are shown in Table 1.

**Table 1:** Baseline characteristics.

	OMPRIS intervention (n = 89)	Waitlist control group (n = 91)
**Socio-demographics**		
Age, years	32.1 (13.0)	31.5 (11.4)
Gender		
Female	20 (22.5%)	21 (23.1%)
Male	69 (77.5%)	67 (73.6%)
Diverse	0 (0.0%)	3 (3.3%)
Marital status		
Unmarried without partner	44 (49.4%)	49 (53.8%)
Unmarried with partner	22 (24.7%)	20 (22.0%)
Married	18 (20.2%)	19 (20.9%)
Divorced	4 (4.5%)	2 (2.2%)
Widowed	1 (1.1%)	1 (1.1%)
Occupational situation		
Full-time employed	30 (33.7%)	22 (24.2%)
Working part-time	4 (4.5%)	4 (4.4%)
Self-employment	2 (2.2%)	6 (6.6%)
Unemployed	7 (7.9%)	12 (13.2%)
Studying at the university	34 (38.2%)	35 (38.5%)
In vocational training	0 (0.0%)	3 (3.3%)
Level of education		
Currently student	4 (4.5%)	3 (3.3%)
High (≥12 years)	80 (89.9%)	75 (82.4%)
Moderate (10 years)	4 (4.5%)	10 (11.0%)
Low (≤9 years)	1 (1.1%)	3 (3.3%)
BFI-10 (Assessment of big five personality traits)		
Neuroticism	6.4 (1.9)	6.7 (2.0)
Extraversion	5.6 (2.0)	5.5 (2.3)
Openness	6.8 (2.2)	7.2 (2.1)
Agreeableness	6.4 (1.6)	6.6 (1.5)
Conscientiousness	5.6 (1.5)	5.4 (1.8)
**Morbidity-related characteristics**		
Ever in treatment because of mental disorders	54 (60.7%)	51 (56.0%)
Comorbidities (current)^a^	32 (36.0%)	32 (35.2%)
Affective disorders (e.g. depression, bipolar disorder)	29 (32.6%)	25 (27.5%)
Eating disorders (e.g. anorexia nervosa, bulimia nervosa, and binge eating disorder^b^)	1 (1.1%)	2 (2.2%)
Neurotic and stress disorders (e.g. panic disorder, social phobia, compulsive disorders)	9 (10.1%)	10 (11.0%)
Suspected ADHD^c^	23 (25.8%)	22 (24.2%)
Comorbidities (past)^a^	13 (14.6%)	19 (20.9%)
Affective disorders (e.g. depression, bipolar disorder)	12 (13.5%)	17 (18.7%)
Psychotic disorder (lifetime)	0 (0.0%)	1 (1.1%)
Mental and behavioural disorders caused by psychotropic substances (e.g. alcohol-, substance-abuse)	1 (1.1%)	2 (2.2%)
AICA-S	12.1 (4.6)	12.6 (5.1)
AICA-SKI:IBS		
Subjectively perceived problematic internet use	8 (9.0%)	11 (12.1%)
Mild disorder	28 (31.5%)	22 (24.2%)
Moderate disorder	36 (40.4%)	40 (44.0%)
Severe disorder	17 (19.1%)	18 (19.8%)
GAF (Global Assessment of Functioning)	69.2 (12.9)	67.8 (12.8)
**Extent and type of addictive behaviour**		
Internet usage weekdays (hrs./day)	6.0 (3.4)	6.0 (3.3)
Internet usage on weekends (hrs./day)	6.5 (3.0)	6.9 (3.5)
Weekly Internet usage (hrs./week)	43.0 (20.81)	43.7 (22.1)
Onset problematic Internet usage since (years)	8.1 (7.0)	7.8 (6.6)
Internet application (most problematically)		
Online streaming	33 (37.1%)	29 (31.9%)
Online gaming	20 (22.5%)	20 (22.0%)
Online pornography	16 (18.0%)	18 (19.8%)
Social networking sites & chatting	12 (13.5%)	16 (17.6%)
Others (online shopping, information research, online gambling, writing emails)	8 (9.0%)	8 (8.8%)

Categorical data are presented as frequencies and percentages. For continuous data, the mean (SD) is given.

a Using the M.I.N.I interview.

b According to WHO's DSM-5 criteria for binge eating disorder.

c Using WHO`s Adult ASRS-V1.1.

**Fig. 2:**
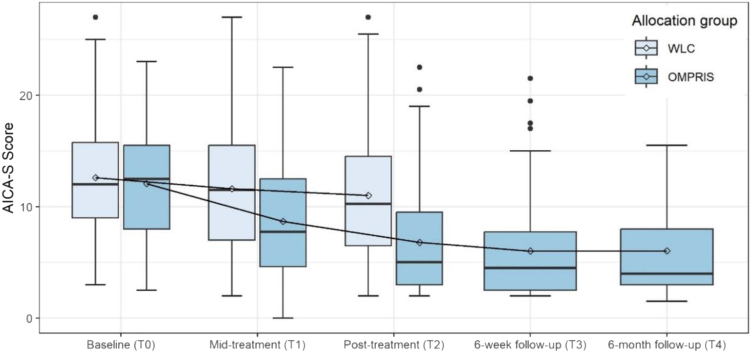
Changes of the Scale for the Assessment of Internet and Computer game Addiction (AICA-S) across measurement points. Comparison of the OMRPIS intervention group with waitlist control (WLC) group. The horizontal line in the center of each box indicates the median, and the point within each box indicates the mean. The upper and lower boundaries of the box mark the 75th and 25th percentiles, respectively. The upper whisker is the maximum value of the data that is within 1.5 times the interquartile range above the 75th percentile. The lower whisker is the minimum value of the data that is within 1.5 times the interquartile range below the 25th percentile. The circles beyond the whiskers are outliers, i.e. all values above 1.5 times the interquartile range above the 75th percentile or all values below 1.5 times the interquartile range below the 25th percentile. T0 indicates time factor, baseline; T1, time factor, mid-treatment; T2, time factor; post-treatment; T3, time factor, 6-week follow-up; and T4, time factor, 6-month follow-up.

The analysis in the intention-to-treat population showed that participants in the OMPRIS intervention group had a significantly greater reduction in AICA-S scores from baseline (mean score 12.1 [SD 4.6]) to post-treatment (6.8 [5.2]) than those in the WLC group (from 12.6 [5.1] to 11.0 [5.4]; estimated mean difference −3.9; [95% CI −5.2 to −2.6]; p < 0.0001; d = 0.92; Fig. 2). Even 6 weeks after intervention (T3 follow-up) and 6 months after randomisation (T4 follow-up), the AICA-S score remained at a level as low as at T2. Sensitivity analyses for the primary outcome are provided in the Supplement.

The number of inconspicuous cases (according to AICA-S score <7.0) in the intervention group could be increased from T0 (n = 12, 13.5%) to T2 (n = 53, 59.6%). The number of addicted cases (according to AICA-S > 13.0) could be reduced from T0 (n = 37, 41.6%) to T2 (n = 12, 13.5%). Thus, 51 out of 81 (62.9%) participants could be downgraded to at least 1 category in symptom severity.

For all secondary outcome scores, the intervention led to a significant effect in a direction advantageous for the participants (Table 2 and eAppendix 6: Figures S1 and S2). In particular, the factors directly associated with internet use showed significant improvement. The intervention group showed a greater reduction in average time spent on the internet (hours/week) from baseline (mean 43.0 [SD 20.8] hrs) to post-treatment (28.1 [15.8] hrs) compared to the WLC group (from 43.7 [22.1] to 38.7 [20.7]; estimated mean difference −11.0; [95% CI −15.0 to −6.1]; p < 0.0001). With regard to motivation for change, the greatest improvements were observed in taking next steps (estimated mean difference 6.1; [95% CI 4.3–7.9]; p < 0.0001).

**Table 2:** Descriptive analysis of baseline pre-treatment (T0) and post-treatment (T2) measurements, change from T0 to T2 and results of the analysis of covariance (ANCOVA).

	Baseline (T0)		Post-treatment (T2)		Change		ANCOVA		
	Control	Inter-vention	Control	Inter-vention	Control	Inter-vention	Coefficient (β)	95% CI	p-value
**Primary outcome score**									
*N*	91	89	88	81	88	81	180		
AICA-S	12.6 (5.1)	12.1 (4.6)	11.0 (5.4)	6.8 (5.2)	−1.5 (4.0)	−5.6 (5.1)	−3.9	[−5.2, −2.6]	<0.0001
**Secondary outcome scores**									
*N*	91	89	88	81	88	81	169		
Weekly internet usage (hrs./week)	43.7 (22.1)	43.0 (20.8)	38.7 (20.7)	28.1 (15.8)	−4.2 (13.7)	−14.7 (21.0)	−11.0	[−15.0, −6.1]	<0.0001
PHQ-9	10.0 (5.6)	9.8 (4.6)	9.8 (5.3)	7.5 (4.9)	−0.2 (4.1)	−2.4 (4.7)	−2.3	[−3.5, −1.1]	0.0002
GAD-7	7.1 (4.8)	7.3 (4.8)	7.1 (5.1)	5.7 (4.4)	0.1 (3.4)	−1.7 (3.9)	−1.7	[−2.7, −0.6]	0.0021
CIUS	35.3 (8.4)	35.1 (8.5)	33.5 (9.7)	26.9 (9.8)	−1.7 (5.0)	−8.5 (10.2)	−6.7	[−9.0, −4.4]	<0.0001
L-1	4.9 (2.2)	4.9 (2.2)	5.3 (2.1)	6.3 (2.1)	0.3 (1.8)	1.4 (1.9)	1.0	[0.5, 1.5]	<0.0001
GSE	25.9 (5.0)	26.1 (5.5)	26.7 (5.2)	28.1 (5.6)	0.8 (3.9)	2.1 (3.4)	1.3	[0.2, 2.3]	0.0224
iSOCRATES									
Ambiguity	15.1 (3.4)	14.7 (4.0)	15.0 (3.47)	13.3 (3.8)	−0.2 (2.8)	−1.3 (3.2)	−1.3	[−2.2, −0.5]	0.0021
Recognition	29.6 (4.6)	29.0 (5.4)	28.5 (4.8)	25.9 (6.4)	−1.2 (3.5)	−3.2 (5.2)	−2.3	[−3.6, −1.0]	0.0007
Taking next Steps	25.2 (7.3)	25.1 (6.6)	25.6 (7.3)	31.3 (6.9)	0.2 (5.5)	6.2 (7.5)	6.1	[4.3, 7.9]	<0.0001

Data are mean (SD) at baseline pre-treatment (T0), follow-up post-treatment (T2) periods, and change from T0 to T2. In the analysis of covariance, the OMPRIS intervention group acts as the reference group. ANCOVA results of the intention-to-treat analysis with LOCF and multiple imputation; CI, Confidence Interval; AICA-S, Scale for the Assessment of Internet and Computer game Addiction; PHQ-9, Patient Health Questionnaire-9; GAD-7, Generalised Anxiety Disorder-7; CIUS, Compulsive Internet Use Scale; L-1, General Life Satisfaction Short Scale; GSE, General self-efficacy scale; iSOCRATES, Stage of Readiness and Treatment Eagerness for Internet use disorder.

The moderator analysis found that the baseline pre-treatment (T0) measurement of the AICA-S (p = 0.079), i.e. the severity of IUD at baseline, age (p = 0.781), gender (p = 0.936) and type of IUD (p = 0.052) are not significant predictors of successful participation in OMPRIS. Due to the trend, however, it cannot be ruled out that the IUD subtypes may influence the therapeutic effect, especially since interaction testing requires larger sample sizes (eAppendix 5, Table S3).

Overall, no participant was excluded due to serious adverse events.

## Discussion

The OMPRIS study is the first high-quality RCT study with sufficient power and sample size to investigate the efficacy of a webcam-based telemedicine treatment for people with IUD. In this project, both more severely affected people and those who still in an early phase of IUD should be addressed. The results of the study showed that the OMPRIS intervention was statistically and clinically effective in reducing IUD symptom severity compared to the WLC group. As a result, our study could thus replicate and confirm what a few pilot studies with small case numbers, less statistical power, or lower intensity of therapeutic intervention had previously suspected.^18^^,^^21^ The OMPRIS study thus complements the existing studies with a waiting list as a control group, because it is the first study to examine a naturalistic telemedical face-2-face treatment using a high-quality RCT. In this way, OMPRIS methodologically extends beyond laboratory-experimental studies (usually with students) and more psycho-educational interventions. Rather, OMPRIS had the character of real face-to-face therapy with a therapist.

The OMPRIS intervention caused a significant reduction in addiction symptoms within a short treatment period of 4 weeks (hypothesis 1). These effects were measurable both in the conservatively measured primary outcome AICA-S and in the somewhat more sensitive and global CIUS. Both scales for the assessment IUD symptoms were described regarding their psychometric parameters as well as their clinical usefulness as recommendable scales that should be used in clinical studies.^44^ Furthermore, the OMRPIS intervention reduced the symptom severity in the OMPRIS group to such an extent that a total of 24 out of 81 (27.0%) participants could be downgraded from the diagnostic category “addicted” to the category “inconspicuous” (according to AICA-S score). Another 27 out of 81 (30.3%) participants improved by at least 1 diagnostic category. Thus, 51 out of 81 (62.9%) participants could be downgraded at least 1 category in the self-reported symptom severity that can be interpreted as a clear clinical significance. Furthermore, sensitivity analyses and the PP analysis also confirmed this significant reduction in symptoms. In addition, the OMPRIS intervention reduced self-reported weekly online time by over 14 h, a reduction of about 34%. These results provide clear evidence that telemedicine treatment via webcam is an effective method of treating patients with IUDs. Follow-up analyses showed that symptom severity did not rebound in the intervention group even 6 months after treatment, again supporting the clinical importance. Additionally, all secondary outcomes showed significant improvement in a direction that is advantageous for the OMPRIS intervention, indicating that the treatments had a positive impact across multiple outcomes. Self-efficacy was also significantly increased through the chosen therapeutic approach, which even lasted for 6 months (hypothesis 2). Motivation to change internet use improved significantly through OMPRIS. In particular, the participants stated that they had taken the first important concrete steps towards healthy internet use (hypothesis 3). This highlights the potential efficacy of the OMPRIS intervention in improving various aspects of the participant's well-being. The effect size of the OMPRIS intervention on reducing IUD symptoms was calculated as d = 0.92, which is a large effect according to Cohen's guidelines.^45^ Further, it is in the range of absolute effect size of psychotherapy for mental disorders described in meta-analyses.^46^^,^^47^ Previous in-person IUD psychotherapy studies in an analogue setting showed effect sizes of g = 0.92–1.51.^9^^,^^12^ The 2019 published STICA study (Short-term Treatment of Internet and Computer Game Addiction) reported an effect size of d = 1.19 for face-to-face group psychotherapy in IUD patients, also using the AICA-S score as an outcome.^10^ However, it should be noted that the STICA population focused only on clinical IUD patients, while our study population included both more severely affected patients and those still in the early stages of IUD. Furthermore, the STICA treatment was significantly more intensive, consisting of 15 weekly in-person group therapy sessions and up to 8 individual sessions.^10^ Compared to the STICA study, the achieved effect size of the short-term OMPRIS intervention is considerable, especially since the low-threshold nature of a telemedical treatment is a significant advantage over a face-to-face setting.

The moderator analysis showed that there were no significant differences between the types of IUD, age, and gender, so there is support that OMPRIS may be effective for all types of IUD, all ages and genders. Due to the statistical trend, however, it cannot be ruled out that IUD subtypes may influence the therapeutic effect. This is especially important since interaction testing requires larger sample sizes. Therefore, further studies focusing on the influence of the IUD subtype require a larger number of cases for each IUD subgroup in order to improve the statistical power of the interaction effect analysis.

OMPRIS significantly improved participants' motivation to change. One could argue that the participants in OMPRIS were the ones who had a high motivation to change anyway. This cannot be ruled out, of course, since the participants actually had to register for OMPRIS themselves. However, almost 42% of participants said they were recommended to participate by parents, medical experts, teachers, or other official bodies. This also indicates that there was external motivation and that not only those who were affected participated but were highly motivated on their own. However, it generally requires either personal interest, psychological strain or external (social) pressure to seek psychotherapeutic treatment.

It should be noted that online therapy for internet addiction may also present some challenges, including technical difficulties such as internet connection problems that can interrupt therapy sessions. Online therapy may also not be suitable for everyone, such as patients with severe mental illness, and relies heavily on self-reported symptoms, making accurate assessment difficult. We have addressed these specifics in our published manual.^31^

This study has a number of strengths. The nationwide recruitment of participants and the fact that it also reached the at-risk population might contribute to the generalisability of the results. The study was sufficiently powered to detect small differences and the diagnostics were conducted in a standardised and comprehensible manner. To our knowledge, this is the first high-quality RCT study to demonstrate the efficacy of webcam-based telemedicine treatment for IUD. The relatively long follow-up and low number of missing data are additional strengths of the study.

A limitation of the study is the absence of an active control group. However, the first aim of this study was to show that a telemedicine intervention is generally effective. This approach, therefore, corresponds to the standard scientific approach and was also chosen in other therapy studies in this field.^10^ According to Grawe,^48^ psychotherapy research can be divided into 4 phases. Phase 1 is called the *legitimation phase* that answers the basic question “Is there a general effectiveness?”, before phase 2 is about testing comparative effects (*competition phase*). Consequently, the OMPRIS study showed the general effectiveness of webcam-based telemedicine treatment for IUD. Further studies should now test the OMPRIS intervention against active control therapies (e.g. analogue CBT). In addition, the waitlist control group corresponds to the actual healthcare situation of patients who usually do not find a therapy due to a lack of motivation or therapy options.

Furthermore, the study was designed in a single-blinded design, meaning that the participants were not masked to intervention allocation. Yet, this is not possible in psychotherapy studies with waitlist control groups. The waiting period, however, was short (4 weeks), so it is not to be expected that bias occurred as a result.

Follow-up measurements were taken after 6 weeks and 6 months to demonstrate that symptom severity did not rebound even 6 months after treatment. Of the 81 participants who were still in treatment at T2, 75 participants gave feedback after 6 weeks and 61 participants after 6 months. So, although there was a drop-out, the response rate after 6 months was still high. This is excellent for an online-based study and even outperforms analogue RCTs in this field.^10^ Furthermore, we did a number of post-hoc sensitivity analyses that supported our main findings. Because of the chosen study design, in which the waiting group also received the intervention after a 4-week waiting period for ethical reasons, the follow-up data of the intervention group could not be compared with those of the waiting group.

In conclusion, our RCT study showed that webcam-based telemedicine treatment of IUD patients is highly effective. The short but intensive online treatment resulted in a significant reduction of symptoms, time spent online, and psychological symptoms. In fact, motivation to change behaviour and self-efficacy could be increased. The reduction of IUD symptoms could still be measured after 6 months. Webcam-based telemedicine therapy expands the availability of accessible and efficient IUD treatment options. The study also highlights the potential for digital health solutions to address behavioural addictions in general, which is an encouraging outcome.

## Contributors

JDH acquired funding and was the principal investigator. LB and JDH were significantly responsible for study coordination/management with support from MP. JDH led the study at all stages with guidance from LB, MP, SH, BtW, KW, PH, AN, BLG, RB, NT, and AN, who helped design the study. JDH wrote the trial protocol with input from LB, MP, NT, AN, KW, BtW, PH and RB. NT, and JB designed and wrote the statistical analysis plan. NT and JB verified the underlying data and did the statistical analysis. NT, JB, and JDH interpreted the analyses. JDH, LB, and MP implemented the study. JDH, LB, MP, SH, KW, MB, BLG, BtW, and PH collected data. JDH, in collaboration with JB, wrote the first draft of the report with support from NT. All authors read, gave critical feedback, and made a revision of the manuscript. All authors approved the final version of the manuscript and took responsibility for its content and the final decision to submit it for publication.

## Data sharing statement

Individual participant data that underlie the results reported in this article can be shared after de-identification (text, tables, figures, and Appendices) by the corresponding author upon reasonable request for academic and research purposes, and subject to Data Sharing Agreements. Researchers who provide a scientifically sound proposal will be allowed to access the de-identified individual participant data. Proposals should be sent to the corresponding author. These proposals will be reviewed and approved by the investigator and collaborators on the basis of scientific merit. To gain access, data requesters will need to sign a data access agreement. The study protocol has already been published and is also available in the supplement. The statistical analysis plan is available in the supplement. Informed consent forms are available on request from the corresponding author.

## Declaration of interests

RB reports that he is CEO (employed) of the Competence Centre of Healthcare Telematics (ZTG Zentrum für Telematik und Telemedizin GmbH). The shareholders of ZTG are various representative bodies of care providers and cost bearers in the German healthcare system. The ZTG receives institutional funding from the state of North Rhine-Westphalia, Germany. Furthermore, RB reports that he is an honorary member of the board of directors of the German Society for Telemedicine (Deutsche Gesellschaft für Telemedizin e.V.).

LB reports that she is on the executive board of the Association of Media Addiction Germany (Fachverband Medienabhängigkeit e.V.), which is committed to creating a network of researchers and practitioners in the German-speaking region within the framework of a large-scale cooperation to deal with internet addictions and gaming disorder. The other authors declare that the research was conducted in the absence of any commercial or financial relationships that could be construed as a potential conflict of interest.

## Appendix A Supplementary data

Supplementary material

Study_protocol_OMPRIS

Statistical Analysis Plan

OMPRIS Study group
