# Supplementary material for: Efficacy of a short-term webcam-based telemedicine treatment of internet use disorders (OMPRIS): a multicentre, prospective, single-blind, randomised, clinical trial
Source: eClinicalMedicine. 2023 Sep 14;64:102216. doi: 10.1016/j.eclinm.2023.102216 (PMC10514435; doi:10.1016/j.eclinm.2023.102216)
Supplement: Supplementary material [file mmc1.docx]

**Efficacy of a short-term, webcam-based telemedicine treatment of internet use disorders (OMPRIS): a multicentre, prospective, single-blind, randomised, clinical trial.**

Jan Dieris-Hirche^1^, Laura Bottel^1^, Jale Basten^2^, Magdalena Pape^1^, Nina Timmesfeld^2^, Bert Theodor te Wildt^3,1^, Birte Linny Geisler^3,4^, Peter Henningsen^4^, Klaus Wölfling^5^, Manfred E Beutel^5^, Anja Neumann^6^, Anja Niemann^6^, Rainer Beckers^7^, Stephan Herpertz^1^, on behalf of the OMPRIS Study Group†

**Online Supplementary Material**

**Table of contents**

**eAppendix 1.** Supplementary Table S1. OMPRIS intervention strategies.

**eAppendix 2.** Supplementary Table S2. Frequencies of mental comorbid disorders measured

with the Mini-International Neuropsychiatric Interview (M.I.N.I.) for DSM-5.

**eAppendix 3.** Per-protocol (PP) analyses.

**eAppendix 4.** Sensitivity analyses.

**eAppendix 5.** Moderator analysis. Supplementary Table S3. Results of moderator analysis.

**eAppendix 6.** Secondary Outcomes. Supplementary Figure S1 & S2. Changes of the secondary outcome scores across measurement points.

**eAppendix 7.** Further OMPRIS Study group members and affiliations.

**eAppendix 1. OMPRIS intervention strategies**

| **Supplementary Table S1.** OMPRIS intervention strategies | | |
| --- | --- | --- |
| **Treatment strategies** | **Treatment phase** | **Key interventions** |
| Motivational interviewing (MI) | All phases | Client-centred approach with empathy and openness |
|  |  | Open questions |
|  |  | Affirmation |
|  |  | Reflective listening |
|  |  | Summarising |
| Cognitive behavioural therapy (CBT) | All phases | Psychoeducation on addiction mechanisms |
|  |  | Self-monitoring of internet use disorder symptoms and assessment of triggers, goal setting, pros and cons, reward mechanisms |
|  |  | Individual model of addiction |
|  |  | Awareness on internet use  Behavioural practices |
|  |  | Strategies to reduce procrastination tendencies |
|  |  | Regulating negative emotions (e.g., aversion and listlessness) |
|  |  | Avoidance changing exposure to cues for IUD behaviour  Self-affirmation  Action planning  Reducing social anxiety |
|  |  | Relapse prevention |
|  |  | Interpersonal skills training |
| Media education | Early and middle phase | Development of media rules and limitations |
| Structuring everyday life | Middle and termination phases | Restructuring of daily routines, sleep hygiene, mealtimes, working hours |
| Social counselling | Middle and termination phases | Help on individual social problems (e.g. unemployment, debt management, housing benefits, assistant living, complying with formalities) |

**eAppendix 2. Frequencies of mental comorbid disorders measured with the Mini-International Neuropsychiatric Interview (M.I.N.I.) for DSM-5**

**Supplementary Table S2.** Frequencies of mental comorbid disorders measured with the Mini-International Neuropsychiatric Interview (M.I.N.I.) for DSM-5. Data are n (%).

| **Diagnosis** | **OMPRIS intervention (n=89)** | **Waitlist control group (n=91)** |
| --- | --- | --- |
| **Comorbidities (current)** |  |  |
| Affective disorders | 29 (32·6 %) | 25 (27·5 %) |
| Major depressive disorder, last two weeks | 4 (4·5 %) | 5 (5·5 %) |
| Major depressive disorder, recurrent | 26 (29·2 %) | 21 (23·1%) |
| Bipolar disorder | 0 (0·0 %) | 0 (0·0 %) |
| Eating disorders | 1 (1·1 %) | 2 (2·2%) |
| Anorexia nervosa | 0 (0·0 %) | 0 (0·0 %) |
| Bulimia nervosa | 0 (0·0 %) | 0 (0·0 %) |
| Binge eating disorder ^a^ | 1 (1·1 %) | 2 (2·2 %) |
| Neurotic and stress disorders | 9 (10·1 %) | 10 (11·0 %) |
| Panic disorder | 2 (2·2 %) | 2 (2·2 %) |
| Agoraphobia | 1 (1·1 %) | 2 (2·2 %) |
| Social anxiety disorder | 4 (4·5 %) | 6 (6·6 %) |
| Obsessive compulsive disorder, last month | 3 (3·4 %) | 1 (1·1 %) |
| Post-traumatic stress disorder | 1 (1·1 %) | 0 (0·0 %) |
| Generalised anxiety disorder | 1 (1·1 %) | 3 (3·3 %) |
| **Suspected ADHD ^b^** | 23 (25·8 %) | 22 (24·2%) |
| **Comorbidities (past)** | 13 (14·6 %) | 19 (20·9 %) |
| Affective disorders | 12 (13·4 %) | 17 (18·7 %) |
| Major depressive disorder, past | 11 (12·4 %) | 16 (17·6 %) |
| Bipolar disorder | 2 (2·2 %) | 0 (0·0 %) |
| Affective disorder with psychotic features | 0 (0·0 %) | 1 (1·1 %) |
| Psychotic disorder (lifetime) | 0 (0·0 %) | 1 (1·1 %) |
| Mental and behavioural disorders caused by psychotropic substances (e.g. alcohol-, substance-abuse) | 1 (1·1 %) | 2 (2·2 %) |

Notes: ^a^ according to WHO’s DSM-5 criteria for binge eating disorder; ^b^ according to WHO`s Adult ASRS-V1.1 for ADHD.

**eAppendix 3. Per-protocol (PP) analyses**

**Results**

In the OMPRIS intervention group all randomised patients participated in at least two online sessions, but 12 patients did not complete the T2 survey by 28 days after release of T2 and are therefore excluded from the PP set. Also in the PP set, the difference between the interventions groups is significant (adjusted p<0·0001; adjusted mean difference -4·1 [95% CI -5·5 to -2·8]).

**eAppendix 4. Sensitivity analyses**

**Results**

On the one hand, the regression weights are unbiased under missing-at-random (MAR) if the factors influencing missingness are part of the regression model. On the other hand, the variability of the imputed data is systematically underestimated. The extent of the underestimation depends on the variance explained and the proportion of missing cases.^1^ Since it can be assumed that there are further factors that have an influence on the missing data, two sensitivity analyses with different replacement strategies, in particular also conservative replacements, were calculated: (1) Participants for whom AICA-S was missing at T1 and T2 were excluded from the analysis, and (2) if AICA-S at time T1 and T2 was missing, score value at time T2 is set to the score value at baseline (T0).

Conservative replacement strategies in the absence of post-treatment measurement (T2) produced similar and also significant results (adjusted p<0·0001 in both sensitivity analyses; adjusted mean difference in sensitivity analysis (1): -3·9 [95% CI -5·2 to -2·6] and sensitivity analysis (2): -3·7 [95% CI -5·0 to -2·5]).

Reference:

1. Little R, Rubin D. Statistical inference with missing data. 2nd ed. New York: Wiley; 2002.

**eAppendix 5. Moderator analysis**

**Supplementary Table S3. Results of moderator analysis.**

|  | **Coefficient** | **95% CI** | **p-value** |
| --- | --- | --- | --- |
| **AICA-S (T0)** | -0·25 | -0·52, 0·03 | 0·079 |
| **Age** | 0·02 | -0·09, 0·12 | 0·781 |
| **Sex** (ref: male) |  |  |  |
| Female | -0·13 | -3·2, 3·0 | 0·936 |
| **Type of IUD** (ref: Online gaming) |  |  | 0·052 |
| Online streaming | 0·85 | -2.7, 4·4 | 0·634 |
| Online pornography | 1·7 | -2·3, 5·7 | 0·412 |
| Social networking sites & chatting | -1·8 | -6·1, 2·4 | 0·399 |
| Other genres (online shopping, information research, online gambling, writing emails) | 6·3 | 1·2, 11 | 0·015 |

**eAppendix 6. Secondary Outcomes**


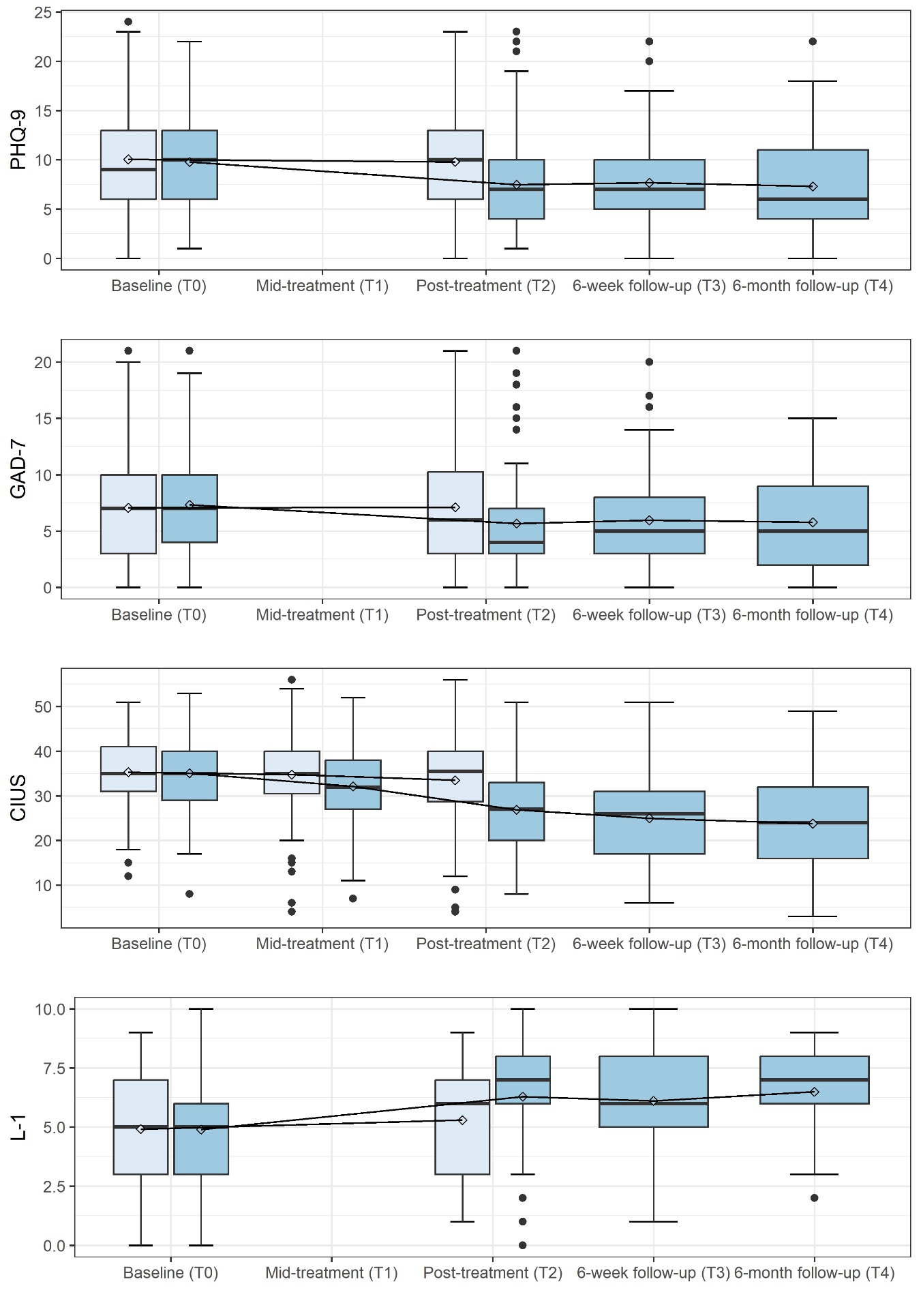
**Supplementary Figure S1.** Changes of the secondary outcome scores across measurement points. Comparison of the OMRPIS intervention group with wait-list control (WLC) group. The horizontal line in the center of each box indicates the median, and the point within each box indicates the mean. The upper and lower boundaries of the box mark the 75th and 25th percentiles, respectively. The upper whisker is the maximum value of the data that is within 1.5 times the interquartile range above the 75th percentile. The lower whisker is the minimum value of the data that is within 1.5 times the interquartile range below the 25th percentile. The circles beyond the whiskers are outliers, i.e., all values above 1.5 times the interquartile range above the 75th percentile or all values below 1.5 times the interquartile range below the 25th percentile. T0 indicates time factor, baseline; T1, time factor, mid-treatment; T2, time factor; post-treatment; T3, time factor, 6-week follow-up; and T4, time factor, 6-month follow-up. PHQ-9, Patient Health Questionnaire-9; GAD-7, Generalised Anxiety Disorder-7; CIUS, Compulsive Internet Use Scale; L-1, General Life Satisfaction Short Scale.


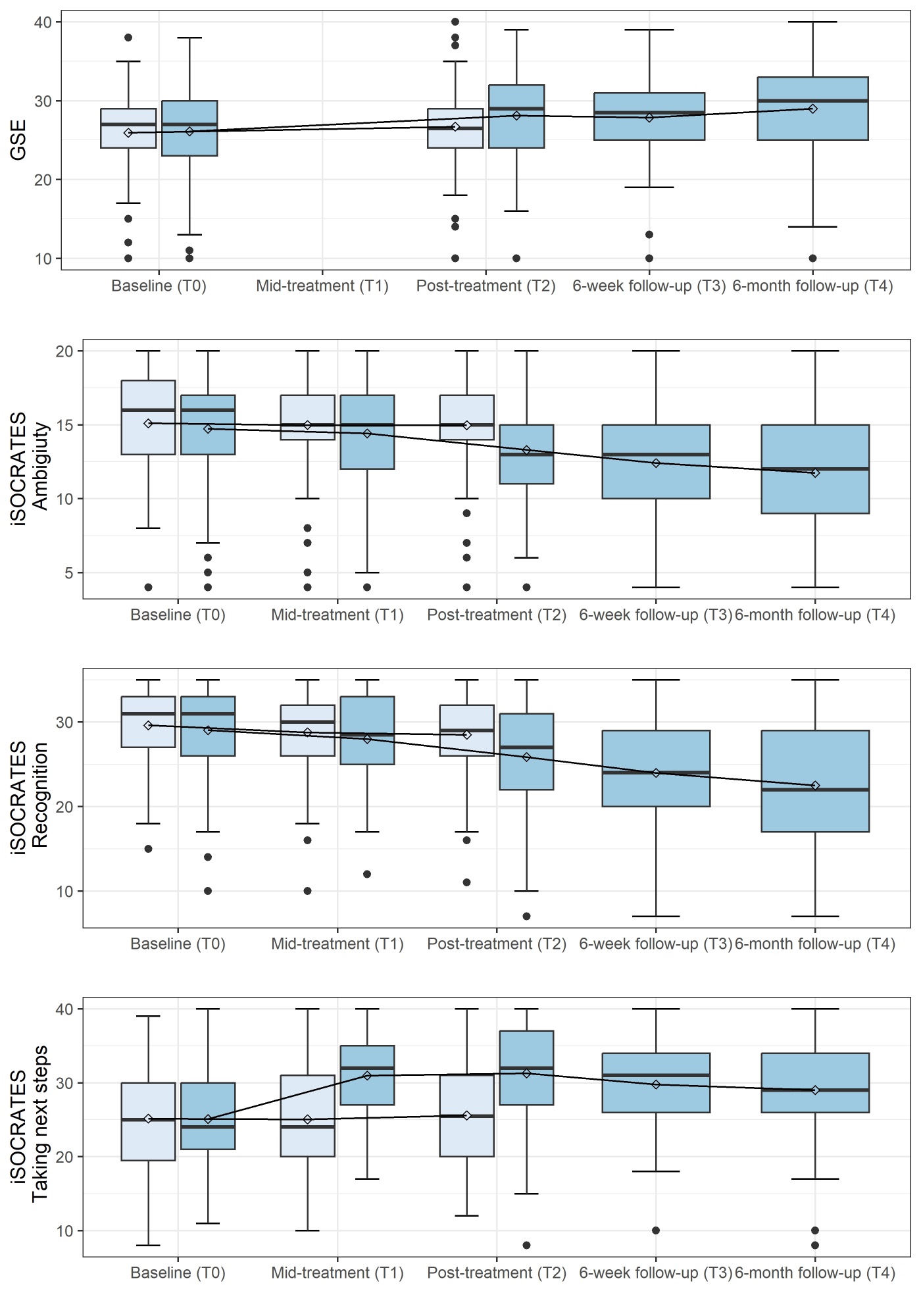


**Supplementary Figure S2.** Changes of the secondary outcome scores across measurement points. Comparison of the OMRPIS intervention group with wait-list control (WLC) group. The horizontal line in the center of each box indicates the median, and the point within each box indicates the mean. The upper and lower boundaries of the box mark the 75th and 25th percentiles, respectively. The upper whisker is the maximum value of the data that is within 1.5 times the interquartile range above the 75th percentile. The lower whisker is the minimum value of the data that is within 1.5 times the interquartile range below the 25th percentile. The circles beyond the whiskers are outliers, i.e., all values above 1.5 times the interquartile range above the 75th percentile or all values below 1.5 times the interquartile range below the 25th percentile. T0 indicates time factor, baseline; T1, time factor, mid-treatment; T2, time factor; post-treatment; T3, time factor, 6-week follow-up; and T4, time factor, 6-month follow-up. GSE, General Self-Efficacy Scale; iSOCRATES, Stage of Readiness and Treatment Eagerness for Internet use disorder.

**eAppendix 7. Further OMPRIS Study group members and affiliations**

Annika Best^7^, Raffaela Böswald^3^, Lorraine Cornelsen^1^, Michael Dreier^4^, Sofie Groen^4^, Alicia Hemmersbach^2^, Vivienne Hillerich^6^, Ina Krahn^1^, Dennis Lowin^7^, Alicia Menze^1^, Silke Neusser^6^, Nehle Penning^1^, Heribert Sattel^5^, Christian Suelmann^7^, Marianne Tokic^2^, Julia Weretecki^1^.

^1^Department of Psychosomatic Medicine and Psychotherapy, LWL-University Hospital, Ruhr University Bochum, Alexandrinenstraße 1-3, 44791, Bochum, Germany.

^2^Department of Medical Informatics, Biometry and Epidemiology, Ruhr University Bochum, Universitätsstraße 105, 44789, Bochum, Germany.

^3^Psychosomatic Hospital Diessen Monastery, Klosterhof 20, 86911, Diessen, Germany.

^4^Outpatient Clinic for Behavioral Addictions, Department of Psychosomatic Medicine and Psychotherapy, University Medical Center of the Johannes Gutenberg-University Mainz, Untere Zahlbacher Str. 8, 55131, Mainz, Germany.

^5^Department of Psychosomatic Medicine and Psychotherapy, University Hospital Rechts der Isar, Technical University Munich, Ismaninger Str. 22, 81675, Munich, Germany.

^6^Institute for Health Care Management and Research, University Duisburg-Essen, Thea-Leymann-Str. 9, 45127, Essen, Germany.

^7^Competence Centre of Healthcare Telematics, Haus Harkorten 8, 58135, Hagen, Germany.
