# Supplementary material for: Efficacy of a short-term webcam-based telemedicine treatment of internet use disorders (OMPRIS): a multicentre, prospective, single-blind, randomised, clinical trial
Source: eClinicalMedicine. 2023 Sep 14;64:102216. doi: 10.1016/j.eclinm.2023.102216 (PMC10514435; doi:10.1016/j.eclinm.2023.102216)
Supplement: Study_protocol_OMPRIS [file mmc2.docx]

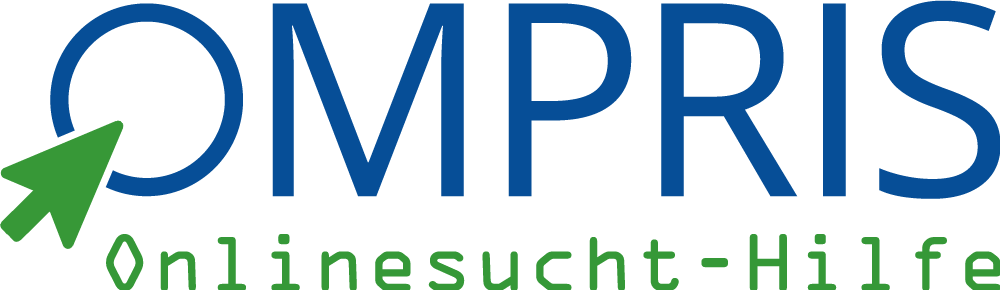


Online-based motivational **program for** the reduction of
problematic media consumption and promoting
motivation in people with
computer game **addiction** and **internet** addiction

(OMPRIS.)

**Study protocol version 4.0 from 3.11.2020**

Funding:

German Innovation Fund of Germany’s Federal Joint Committee (G-BA), grant number 01VSF18043.

Consortium leadership:

Jan Dieris-Hirche, MD

LWL University Hospital of the Ruhr University Bochum, Department of Psychosomatic Medicine and Psychotherapy

LWL University Hospital, Ruhr University Bochum

Alexandrinenstr. 1-3

44791 Bochum

Germany

Jan.dieris-hirche@rub.de


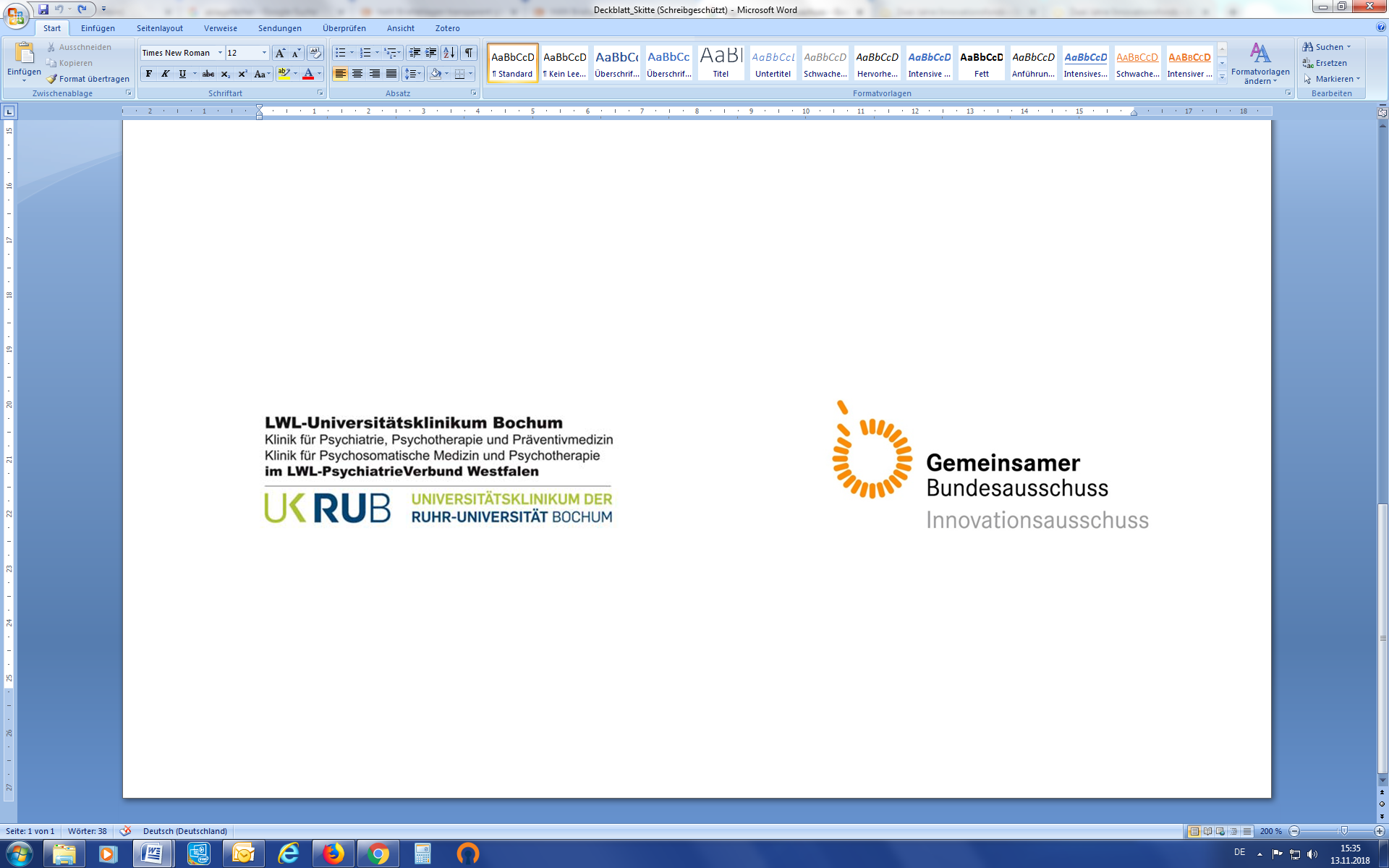


1 Synopsis

| **General information about the project** | |
| --- | --- |
| **(Overall) project management or consortium management/** **Applicant** | Consortium leader, project management and applicant: Jan Dieris-Hirche, MD, Tel.: +49(0)234-5077-3135, Fax: +49(0)234-5077-3759. Email: jan.dieris-hirche@rub.de  Consortium leader, director of the clinic: Prof. Dr. med. Stephan Herpertz, Tel.: Tel.: +49(0)234-5077-3100, Fax: +49(0)234-5077-3111. Email: stephan.herpertz@rub.de. Both active at LWL-University hospital, Ruhr-University Bochum, Department of Psychosomatic Medicine and Psychotherapy, Alexandrinenstraße 1-3, 44791 Bochum, Germany |
| **Participating institutions/ consortium partners (Kp)/ cooperation partners (Ko)**  R=Recruitment  T=treatment  E= Evaluation  B=Biometry | Kp: 1.) TU München, Klinikum rechts der Isar, Klinik u. Poliklinik für Psychosomatische Medizin und Psychotherapie (R,T), 2.) Psychosomatische Klinik Kloster Dießen (R,T), 3.) Universitätsmedizin Johannes Gutenberg-Universität Mainz, Klinik für Psychosomatische Medizin und Psychotherapie (R,T), 4.) Department of Medical Management, Faculty of Economics, University of Duisburg-Essen, Essen Campus (B,E), 5.) Department of Medical Informatics, Biometry and Epidemiology, Ruhr University Bochum (B,E), 6.) ZTG Zentrum für Telematik und Telemedizin GmbH Bochum (concepts), Ko: Fachverband für Medienabhängigkeit e.V. (Network) |
| **Project title/acronym** | Online-based motivational program to reduce problematic media consumption and promote treatment motivation in people with computer game addiction and internet addiction / OMPRIS |
| **Research field/** **Disease/** **Target population** | Internet-related disorders/ DSM-5: Internet Gaming Disorder, ICD-11: Gaming Disorder, Harmful Use of the Internet (ICD-11: Hazardous Gaming)/ Affected persons ≥ 16 years of age with perceived distress and/or problematic use of the Internet or Internet addiction. |
| **Project goals, hypothesis** | Development of a new care structure for people with problematic media consumption and internet-related disorders.  **Primary hypotheses:**   1. Participation in OMPRIS leads to a reduction of media addiction symptoms, i.e. problematic media use behavior, in the participants.   **Secondary hypotheses:**   1. Participation in OMPRIS leads to an increase in the participants' motivation to change with regard to problematic media use. 2. Participation in OMPRIS leads to an increase in quality of life for the participants. 3. Participation in OMPRIS leads to a reduction in depression and anxiety among participants. 4. Participation in OMPRIS leads to an increase in social functioning for participants. 5. Exploratively, it will be investigated whether there are possible predictors (e.g. person-related characteristics) for successful participation in OMPRIS. 6. The referral successes in analogue treatment offers are surveyed. 7. There is a subjectively experienced impact of media consumption due to the COVID-19 pandemic. |
| **Study design and** **Methodology** | Multicentre, prospective, randomised controlled trial (RCT) with a waiting control group. Analyses of clinical outcome variables and health economic analyses. |
| **Intervention/measure/**  **Object of investigation** | Webcam-based diagnostics & intervention (4 weeks) in individual setting. Subject of investigation: Problematic media use behaviour/addiction symptoms, motivation to change, QoL, psychological distress, (in-)direct costs, social functioning. |
| **Study population and sample size** | Patients with problematic internet use or internet addiction. Sample estimate: n= 81 patients are to be recruited per group, a total of 162 patients on the basis of a preliminary study and experiences of the treatment centres Bochum & Mainz. |
| **Region of the project** | Germany-wide online-based guided, structured, individual counselling; 3 treatment centres (Bochum, Mainz, Dießen/Munich). |
| **Data basis** | Primary data through patient survey: Questionnaires (self-report), structured clinical interviews, (in-) direct medical costs, socio-demographic data. |
| **Data analysis** | Primary outcome: addiction symptoms/problematic media use. Secondary: motivation to change, depressive & anxiety symptoms, social function, quality of life, cost of illness, resource use, statistical analysis (ITT) for all continuous endpoints (especially the primary) covariance analyses. |
| **Requested funding period** | 3 years (36 months) |
| **Funding applied for** | 1.309.985,21 € |
| **Funding** | German Innovation Fund of Germany’s Federal Joint Committee (G-BA), grant number 01VSF18043. |
| **Registration in a publicly accessible study register** | The study protocol is registered with the DRKS under the number ID: DRKS00019925, Date of registration: 13.03.2020. |

2 Responsibilities

#### 2.1 Responsible persons/participants

| **Name** | **Institution** | **Phone, Fax, E-Mail** | **Responsibility/Role** |
| --- | --- | --- | --- |
| Univ.-Prof. Dr. med. Stephan Herpertz | LWL University Hospital of the Ruhr University Bochum, Department of Psychosomatic Medicine and Psychotherapy | Phone: 0234-5077-3110  Fax: 0234-5077-3111  stephan.herpert@rub.de | Consortium leadership/ Director of the clinic |
| Jan Dieris-Hirche, MD | LWL University Hospital of the Ruhr University Bochum, Department of Psychosomatic Medicine and Psychotherapy | Phone: 0234-5077-3135  Fax: 0234-5077-3759  [jan.dieris-hirche@rub.de](mailto:jan.dieris-hirche@rub.de) | Consortium management/project management/supply/administration/planning |
| PD Dr. med. Bert te Wildt | Psychosomatic Clinic Kloster Dießen / Ruhr University Bochum | Phone: 08807 2251-610  Fax: 08807 2251-240  [bert.tewildt@artemed.de](mailto:bert.tewildt@artemed.de) | Consortium partners/concept development/supply |
| Univ.-Prof. Dr. med. Peter Henningsen | Clinic and Polyclinic for Psychosomatic Medicine and Psychotherapy, Klinikum rechts der Isar, Technical University of Munich | Tel.: 089 4140-4311  Fax: 089 4140-4315  P.Henningsen@tum.de | Consortium partner/supply |
| Dr. sc. hum. Klaus Wölfling | ^5^Outpatient Clinic for Behavioral Addictions, Department of Psychosomatic Medicine and Psychotherapy, University Medical Center of the Johannes Gutenberg-University Mainz | Tel: 06131 17-6147  Fax: 06131 17-6439  woelfling@uni-mainz.de | Consortium partner/supply |
| PD Dr. med. Dr. rer. pol. Anja Neumann &  Dr PH Silke Neusser | Institute for Health Care Management and Research, University Duisburg-Essen | Tel.: +49 201 183-4077  Fax: +49 201 183-4073  [anja.neumann@medman.uni.due.de](mailto:anja.neumann@medman.uni.due.de) | Consortium partners/health economic evaluation/biometrics |
| Prof. Dr. Nina Timmesfeld & Prof. Dr. rer. nat. Hans Trampisch (emer.) | Ruhr University Bochum  Department of Medical Informatics, Biometry and Epidemiology | Phone: +49 234 32 27790  Fax: +49 234 32 14325  [hans.J.Trampisch@ruhr-uni-bochum.de](mailto:hans.J.Trampisch@ruhr-uni-bochum.de)  nina.timmesfeld@rub.de | Consortium partners/clinical evaluation/biometrics |
| Rainer Beckers M.P.H., M.A. | ZTG Centre for Telematics and Telemedicine GmbH | T +49 234 97 35 170  F +49 234 97 35 1730  [info@ztg-nrw.de](http://ztg-nrw.de) | Consortium partners/online usability concepts/networking strategies |
| Dr. Kai Müller, 1st Chairman of Fachverband Medienabhängigkeit e.V. | Fachverband Medienabhängigkeit e.V. | Tel.: 0251 591-4837  Fax: 0251 591-5484  [m.wirtz@fv-medienabhaengigkeit.de](mailto:m.wirtz@fv-medienabhaengigkeit.de)  [markus.wirtz@lwl.org](mailto:markus.wirtz@lwl.org) | Cooperation partner/network |

3 State of research and derivation of the research questions

**3.1 Scientific background**

Since the release of the internet in 1993, there has been a rapid and steadily increasing social spread and use. In 2017, 81% of all Germans were online (D21 Digital Index 2017 / 2018). However, parallel to the revolutionary development of the internet, internet-associated disorder patterns of behavioural addictions also emerged, which have now been researched for 20 years. In 2013, Internet Gaming Disorder with the specific addiction criteria (Fig. 1) was included as a research diagnosis in the appendix of the Diagnostic and Statistical Manual of Mental Disorders (DSM-5) for the first time. In addition, the World Health Organization (WHO) recently published the inclusion of the diagnosis of (Internet) Gaming Disorder in the chapter "Disorders based on addictive behaviour" in its advance notice of the 11th version of the International Statistical Classification of Diseases and Related Health Problems (ICD-11 Beta-Draft, 2017). The development of a first AWMF S1 guideline for the treatment of people with internet-related disorders is planned. Internet and computer game addiction are also evident in the medical care and health insurance system. The DAK Gesundheit study published in 2015 reported an increasing prevalence (5% pathological use, 11% harmful use with loss of control) for pathological internet use in the sense of an addictive disorder among young people (DAK, 2015). This development led to the first-time inclusion of computer game addiction and internet addiction in the Federal Government's Drug and Addiction Report 2017 (Federal Government Commissioner on Narcotic Drugs, 2017). The 2015 drug affinity study of the BZgA also showed high and, compared to 2011, growing prevalence for computer game and internet addiction (5.8% of 12-17 year old adolescents and 2.8% of 18-25 year old adults, respectively) with weekly usage times of 21 and 22 hours (Orth, 2017). The representative PINTA study (Prevalence of Internet Addiction), commissioned by the German Federal Ministry of Health (BMG) in 2011 and post-evaluated in 2013, showed that about 550,000 people (1.5%) between the ages of 14 and 64 are affected by internet addiction throughout Germany. Both sexes were affected almost equally often. Younger participants showed higher prevalence for internet addiction (up to 4.0%). The estimate for harmful internet use was 4.6% of the total sample (Bischof et al., 2011).

Figure 1: DSM-5 criteria for Internet Gaming Disorder

**DSM-5 criteria for Internet Gaming Disorder:**

**Persistent and repeated use of the internet for the** purpose of **online gaming**, often with other gamers, resulting in clinically significant disability or limitations, characterised by **five (or more) of** the following symptoms over a period of **12 months** duration:

1. **Continuous preoccupation** with internet or online games. (The affected person thinks about previous online games or is mentally preoccupied with future games. Online games become the dominant activity of daily life).
2. **Withdrawal symptoms** when online gaming is not available. (These withdrawal symptoms are typically described as irritability, anxiety or sadness. Physical symptoms in the sense of drug withdrawal are not described).
3. **Development of tolerance** with the need to spend increasing amounts of time playing online games.
4. **Unsuccessful attempts to** stop participating in online gaming.
5. **Loss of interest in** previous hobbies or activities as a result of online gaming.
6. Continued excessive online gaming **despite knowledge of the** psychosocial problems.
7. **Deceiving** family members, therapists or others about the true extent of online gambling.
8. Use of online games to get out of or relieve **negative emotions** (such as feelings of helplessness, guilt or anxiety).
9. **Jeopardising or losing** important acquaintances, job, education or career opportunities because of online gaming.

People with internet or computer game addiction usually have mental comorbidities with high psychosocial distress and global functional impairment, although causality has not yet been proven. The most common comorbid disorders are depression, social phobias/anxiety disorders, ADHD and personality disorders (Lee et al., 2014). Risk factors are considered to be male gender, young age, low social competence, impulsivity and aggressiveness, loneliness, high internet usage time, problem procrastination and avoidance behaviour. Social competence, self-confidence and social integration are considered protective factors (Mihara & Higuchi, 2017). Longitudinal studies show temporal stability of computer game and internet addiction after 6 months (Lemmens et al., 2011) and 2 years (Brunborg et al., 2014), although the overall findings are not consistent (Mihara & Higuchi, 2017). Brain imaging studies also confirm the model of behavioural addiction in terms of structural and functional changes (Weinstein, 2017). The still incomplete care situation for the specific treatment of people with computer game and internet addiction as well as the addiction-specific motivation problems currently lead to an underuse of those affected, who usually come into treatment very late - often under pressure from the social system. This leads to chronic courses of the disease, in which there can be clear social functional deficits (loss of education or job, social isolation, neglect). If those affected seek psychotherapeutic treatment, it is usually the psychological comorbidities (depression, ADHD, anxiety disorders) that are treated; the internet or computer game addiction usually remains insufficiently treated due to a lack of disorder-oriented therapy approaches. In addition, the harmful use of the internet, but also internet addiction, is still played down socially and therapeutically. A possible early, (secondary) preventive intervention is thus often considered too late.

The current social Corona pandemic also influences media use in the professional and private life environment, so that there are increased media use times (e.g. DAK Study 2020). Within the framework of the OMPRIS project, possible subjectively experienced influences of the Corona pandemic on media use are therefore also to be surveyed. The above derivation results in the following project goals, which are to be worked on within the framework of the OMPRIS study:

**3.2 OMPRIS project objectives:**

1) Development of a low-threshold and early applicable online-based guided, structured, manualised individual treatment to "pick up" affected and endangered people in the "space of addiction" internet and, if necessary, to transfer them to analogue treatment offers.

2) Enhancement of motivation to change and behaviour using a compact, telemedical, guided, structured, manualised individual treatment to reduce internet addiction symptoms and secondary prevention of further addiction problems.

**3.3 Working hypotheses:**

**Primary hypotheses:**

1. Participation in OMPRIS leads to a reduction of internet use disorder symptoms, i.e. problematic media use behaviour.

**Secondary hypotheses:**

1. Participation in OMPRIS leads to an increase in the participants' motivation to change with regard to problematic media use.
2. Participation in OMPRIS leads to an increase in quality of life for the participants.
3. Participation in OMPRIS leads to a reduction in depression and anxiety among participants.
4. Participation in OMPRIS leads to an increase in social functioning for participants.
5. Exploratively, it will be investigated whether there are possible predictors (e.g. person-related characteristics) for successful participation in OMPRIS.
6. The referral successes in analogue treatment offers are surveyed.
7. The influence of the current Corona pandemic on problematic media use is surveyed.

4. Methodology and implementation

**4.1 Study design:**

The multicentre OMPRIS study is planned as a randomised controlled trial (RCT) in a waiting group control design (Fig.2). The study is coordinated by the LWL University Hospital for Psychosomatic Medicine and Psychotherapy of the Ruhr University Bochum (Centre West). Three other clinics (Centrum Mid: Mainz, Centrum South: Dießen & Munich) will participate in the recruitment and counselling of participants. The evaluations and statistical analyses are carried out by the Department of Medical Informatics, Biometry and Epidemiology at the Ruhr University Bochum and the Chair of Medical Management, Faculty of Economics at the University of Duisburg-Essen, Essen Campus. The development of the online environment as well as the online-related strategies (positioning etc.) are coordinated by the ZTG (Zentrum für Telematik und Telemedizin GmbH). First, a baseline survey T0 will take place. All participants in the study will then be randomly assigned to either the intervention group (IG, 4 weeks) that starts immediately or to a waiting control group (KG, 4 weeks). In order to keep the drop-out rate low and to record the effect of the first and last two weeks of the intervention, an email is sent out to the intervention group (IG) and the waiting group (KG) after two weeks (half-time of the intervention) with current information, such as the duration until the start of the intervention and the request to complete a few questionnaires on symptom severity and motivation (T1) for monitoring the course. After completion of the OMPRIS intervention (IG) or the waiting period (KG), the T2 survey (post-treatment) takes place. Completion of the post-treatment measurement is rewarded with 20 euros per participant. Afterwards, the control group starts the OMPRIS intervention. 6 weeks after each intervention, the participants of the respective groups will be invited to participate in the follow-up survey (T3a/b). In addition, a second follow-up survey will be conducted after 6 months by email (T4). In order to keep the response rate to the follow-up surveys as high as possible, all participants will receive a renewed allowance of 15 euros each after the follow-up survey. In total, a maximum allowance of 50 euros would be possible if all questionnaires were completed at all times.

**4.2 Access to studies via the internet:**

Via an informative homepage freely available on the internet [(](http://www.onlinesucht-hilfe.com)www.onlinesucht-hilfe.com), all interested persons with internet access are first invited to participate in a short screening (standardised questionnaire OSV-S, see below) to assess problematic or addictive internet-associated usage behaviour. Via the homepage, participants are also offered detailed information and illustrations on the study structure as well as an illustration of the design. In the case of an OSV-S score≥ 7 (indication of problematic use), a direct invitation to participate in the OMPRIS intervention is issued. A detailed participant education takes place via webcam, in which information is provided about the purpose and procedure of the study and the inclusion and exclusion criteria for participation in OMPRIS are reviewed in detail. In the case of an inconspicuous OSV-S score, a media educational training text on the prevention of internet addiction is provided. Participants with an inconspicuous OSV-S score can still take part in the OMPRIS intervention if they wish, as the offer also has a preventive effect. The OMPRIS project is presented to the widest possible audience of potentially affected persons, their relatives and professionals through intensive press work and networking (e.g. specialised agencies, professional associations, etc.) in order to achieve sufficient recruitment.

**4.3 Procedure for informing and obtaining consent**

The participants are informed and explained about the purpose of the study in a webcam-based individual interview. In the course of this conversation, the detailed procedure of the study is explained and the decisive aspects of data storage are explained. If there are any questions or open points on the part of the participants, these can also be answered and discussed during the participant education interview. The inclusion, discontinuation and exclusion criteria are recorded in a standardised manner before participation in the intervention in the course of the telemedical informed consent interview, which is carried out by a trained psychologist via webcam. For this purpose, a structured clinical interview for the assessment of mental disorders (MINI) is carried out within the framework of the informed consent interview, which records the psychiatric diagnoses of the exclusion criteria. The interview also explicitly asks about known previous somatic diseases that could themselves be associated with an impulse control disorder, or whose treatment with hormone-influencing drugs could be associated with an impulse control disorder (e.g. Parkinson's disease with dopaminergic medication). In addition, the known previous psychiatric illnesses and the current psychiatric or psychotherapeutic treatment are asked about in the information session. If a person is already undergoing psychiatric or psychotherapeutic treatment for media addiction, this is considered an exclusion criterion, as the intervention is not intended to replace medical treatment for computer game addiction. The participants are made aware of the exclusion criteria in the written informed consent. After discussing the consent form together and checking the inclusion and exclusion criteria, the participants are asked to agree to the consent form after the interview. For the participation of minors between the ages of 16 and 18 (as a relevant target group), the consent of the participant and a parent or guardian is required, as well as the participation of the parent or guardian in the informed consent interview. Participants between 16 and 18 years of age are informed in the presence of a parent via webcam. Consent to participate is then confirmed separately by the parent and the person concerned via the OMPRIS portal. Participants and parents receive all information directly as a pdf file via the OMPRIS portal. The written consent of the person concerned or parent/guardian must be uploaded via the study platform.


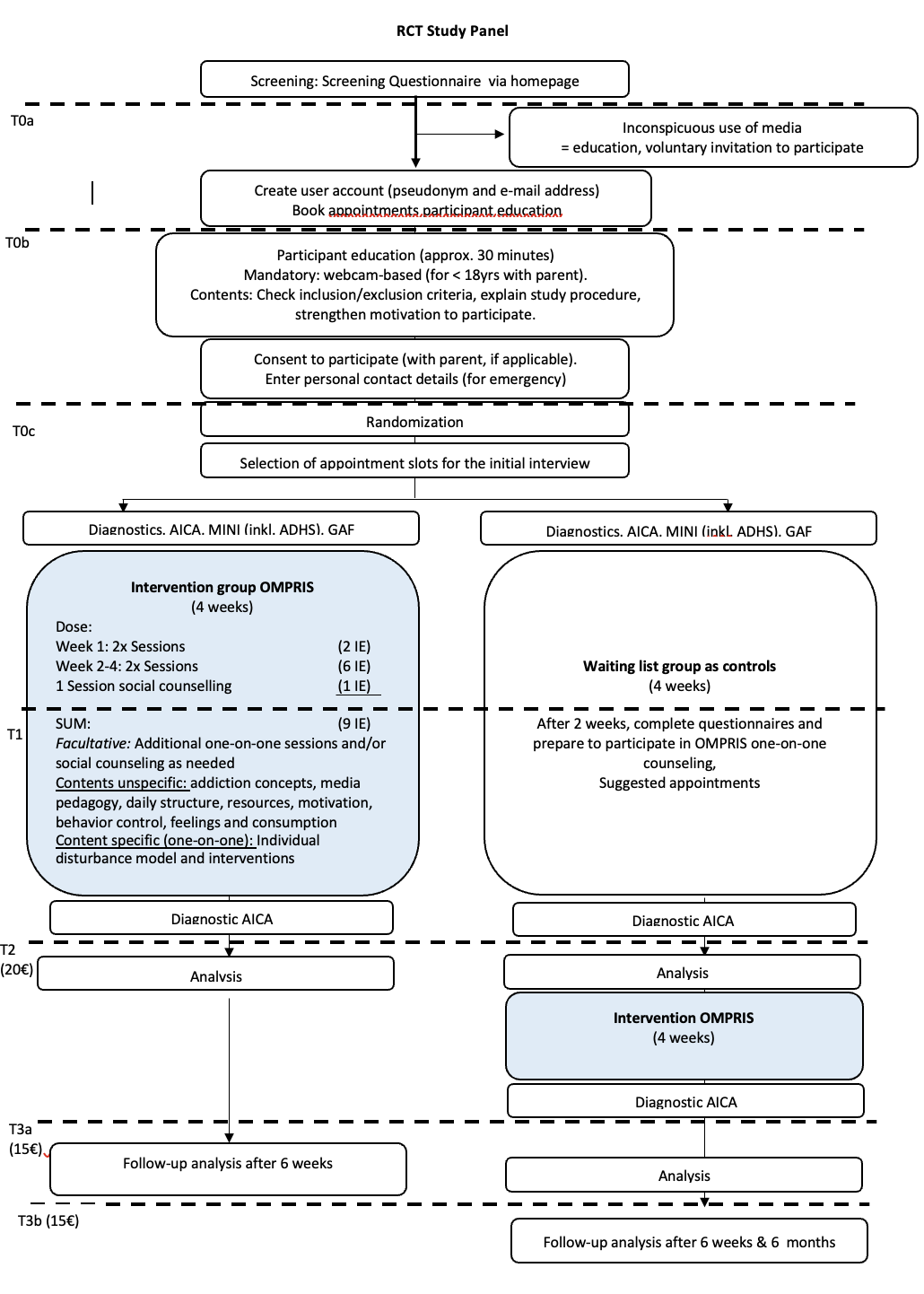


Fig. 2: Flow chart of the study in detail.

**4.4 Study population:**

The primary study population of the OMPRIS study are those affected by problematic or pathological use of computer games, other specific types of internet use (internet pornography, social networking sites, etc.) or the internet in general. However, in order to promote the preventive approach of the intervention, subjects/participants who have achieved an unremarkable score in the screening are also admitted on request. These are evaluated as a subgroup in the secondary analysis. Broad inclusion criteria were chosen to enable the lowest possible threshold for participation:

1. Possibility of regular use of an internet connection with webcam and/or at least microphone as well as an email address
2. Age from 16 years (under 18 years with consent of at least one parent in the form of a short written consent (via photo/email) and a short webcam-based confirmation).
3. Sufficient understanding and speaking of the German language
4. Consent to de-pseudonymisation in case of emergency (e.g. acute suicidality in the context of counselling sessions).

The exclusion criteria were defined as:

1. Acute psychotic symptoms (acute delusions, hallucinations, massive thought disorders)
2. Acute suicidal tendencies or self-endangerment
3. Severe intelligence impairment that prevents participation
4. Substance dependence in the foreground
5. Age under 16 years or lack of consent of the parent/guardian in the case of minors.
6. Lack of German language skills
7. Lack of regular internet access with webcam as well as lack of email address.
8. Presence of known somatic pre-existing diseases which themselves or their treatment with hormone-influencing drugs could be associated with an impulse control disorder (e.g. Parkinson's disease with dopaminergic medication).
9. Currently undergoing psychiatric or psychotherapeutic treatment for media addiction, as the OMPRIS intervention is not intended to replace medical treatment for computer game addiction.

Termination criteria:

1. At the participant's own request
2. Occurrence/recognition of exclusion criteria (see III.10.)
3. If, in the opinion of the study director/advisor, remaining in the study would be detrimental to the welfare of the study participant
4. Serious adverse event associated with the investigational method used.
5. If, in the opinion of the study director, protocol violations by the study participant result in erroneous data.

**4.5 Variables and survey instruments used:**

Primary outcome variable:

1. Measures of improvement, i.e. *reduction of* **internet-related addiction symptoms** measured with the Scale of Online Addictive Behaviour in Adults (OSV-S, Wölfling et al., 2010). The OSV-S is a 15-item scale for measuring online addictive behaviour in adults. The scale covers all relevant addiction criteria oriented to the DSM-5 and shows very good psychometric properties. The questions are answered on a five-point Likert scale from 0 = "never" to 4 = "very often". The English version of the questionnaire is called AICA-S.

Secondary outcome variables and other measures/variables:

1. Measures of the improvement of a **motivation to change**, measured with the internet-related version of the Stages of Change Readiness and Treatment Eagerness Scale (iSOCRATES-D). This is a specially developed German version of the SOCRATES scale established in addiction medicine (Miller & Tonigan, 1996), adapted for internet addiction. The 19 questions are answered on a five-point Likert scale from 1 = "do not agree" to 5 = "completely agree".
2. Reduction of clinical **depressiveness and anxiety** measured with the relevant questions (PHQ-9 and GAD-7) of the Patient Health Questionnaire (PHQ-D, Löwe et al., 2002). This is an established psychodiagnostics instrument for screening and case identification as well as the severity of the most common mental disorders. The PHQ-D is standardised and validated with regard to its implementation and evaluation.
3. Single-item scale to assess **general life satisfaction** (L-1; Beierlein, Kovaleva, & László, 2014). The scale contains only one item "How satisfied are you, all in all, with your life at present?", which is answered on an 11-point scale from "not at all satisfied" (0) to "completely satisfied" (10).
4. **The general self-efficacy expectancy (SWE)** scale involves answering 10 items on a four-point scale from "not true" to "true exactly". It measures optimistic competence expectancy, i.e. the confidence in mastering a difficult situation, whereby success is attributed to one's own competence (Schwarzer & Jerusalem, 1999).
5. The 10 Item **Big Five Inventory Short Scale** (BFI-10, Rammstedt et al., 2013) is used to assess the five **dimensions of personality** according to the BIGS-5 model. BFI-10 was validated on a large, population-representative sample. The results demonstrate satisfactory psychometric parameters for the BFI-10. In addition, the construct and criterion validity of the procedure could be empirically supported.
6. Improvement in **health-related quality of life** measured with the EQ5D (EuroQol Group, 1990). The questionnaire expresses health status in a one-dimensional measure from 0 (very poor) to 1 (very good).
7. **Global** Assessment of Functioning (GAF). GAF is used to assess a person's general level of functioning. Physical illnesses and environmental conditions are explicitly not to be taken into account. Mental, social and occupational functioning are thought of on a hypothetical continuum from mental health to illness. The scale is used within the fourth edition of *the Diagnostic and Statistical Manual of Mental Disorders* [(DSM-IV)](https://de.wikipedia.org/wiki/Diagnostic_and_Statistical_Manual_of_Mental_Disorders#Multiaxiale_Einteilung) and within the [Operationalised Psychodynamic Diagnostic (OPD)](https://de.wikipedia.org/wiki/Operationalisierte_Psychodynamische_Diagnostik#Achse_I_-_Krankheitserleben_und_Behandlungsvoraussetzungen). The assessment period in OPD 2 refers to the last 7 days. The GAF scale was originally developed by the [American Psychiatric Association](https://de.wikipedia.org/wiki/American_Psychiatric_Association) in 1989 and is divided into 10 levels of functioning, each with 10 items. It ranges from 100 (highest level of functioning) to 1 (lowest level of functioning).
8. The quality of the therapeutic relationship is assessed by means of the **Helping Alliance Questionnaire** (HAQ) for participants (Bassler et al., 1995). The HAQ captures characteristics of the [therapeutic relationship](https://portal.hogrefe.com/dorsch/helping-alliance-questionnaire-haq/therapiebeziehung-1/), can be used especially for therapy evaluation (process and outcome; [psychotherapy research](https://portal.hogrefe.com/dorsch/helping-alliance-questionnaire-haq/psychotherapieforschung-1/)) and comprises 12 items with a 6-step response format. A total value and two subscale values, *relationship satisfaction of the participants* and *success satisfaction of* the participants, can be calculated.
9. Subjectively experienced influence of the **Corona pandemic on media consumption** (9 questions for participants, 5 questions for counsellors, self-designed) and subjective experience of stress due to the Corona pandemic (Fear of Covid-19 scale, Ahorsu et al. 2020), 7 items.

Structured clinical interviews:

1. Elicitation of symptoms of **Internet-related disorder** (at the beginning and at the end of the intervention) evaluated by the **structured clinical interview on Internet-related disorders** (AICA-SKI:IBS, Müller & Wölfling, 2017). The interview is oriented towards the DSM-5 criteria for Internet Gaming Disorder and provides a guided exploration of the main symptoms of Internet-related addiction. It was developed and validated on a sample of 500 outpatients.
2. Presence of **mental comorbidities or relevant exclusion criteria** (Axis I disorders) assessed with structured diagnostic **brief interview for mental disorders MINI 6.0** (International Neuropsychiatric Interview, Sheehan et al., 2010), a structured clinical interview based on the criteria of the DSM.

Other variables collected:

1. Need / number of **placements in analogue settings**.
2. **Direct and indirect costs of illness** per person affected in standard care.
3. A **media history** with relevant internet-associated biographical questions.
4. **Socio-demographic** information about the person.
5. **Lifestyle parameters** (sleep, food, sport, etc.).
6. Medical history regarding **previous somatic and mental illnesses and treatments,** medication.

| Tab. 1: Instruments and clinical interviews | |  |
| --- | --- | --- |
| **Instrument** | **Construct** | **Items** |
| OSV-S | Online addictive behaviour | 15 |
| iSOCRATES | Motivation for change | 19 |
| PHQ-9 | Depressiveness | 9 |
| GAD-7 | Anxiousness | 7 |
| L-1 | Life satisfaction | 1 |
| SWE | Self-efficacy | 10 |
| BFI-10 | Personality patterns | 10 |
| EQ5D | Health-related quality of life | 5 |
| GAF | Global functional level | 1 |
| HAQ | Therapeutic relationship | 12 |
| FCV-19S | Covid-19 stress | 7 |
|  |  |  |
|  | Indirect / direct costs | 10 |
|  | Media amnesia | 2 |
|  | Lyfestyle Prameter | 12 |
|  | Sociodemographic data | 7 |
|  | Pre-existing conditions / treatments | 10 |
|  | Evaluation OMPRIS | 10 |
|  | COVID & Media Consumption (Consultant) | 5 |
|  | COVID & Media Consumption (Participants) | 9 |
|  |  |  |
| Structured interviews | AICA Addiction Criteria DSM-5 |  |
|  | Mental comorbidities MINI 6.0.0 |  |

**4.6 Intervention in detail:**

The completely webcam-based OMPRIS intervention offers a manualised treatment approach with specifically selected interventions from different media pedagogical and psychotherapeutic directions. Based on many years of clinical experience as well as the STICA study (Jäger et al. 2009), behavioural therapy, addiction therapy and everyday structuring, resource-promoting interventions are offered (Table 2). In addition, social work assistance is offered to overcome socio-medical problems. The basic counselling approach is based on the motivational interviewing approach established in addiction medicine (see below). A conceptual draft of the OMPRIS manual is being prepared separately. The OMPRIS intervention offers a total of about 9 intervention units over a period of 4 weeks. Key interventions will be delivered in webcam-based one-to-one consultations. Figure 2 shows the overview of the general process of the project, which can be divided into several stages:

1. Completion of a short self-test to assess internet-related addiction symptoms (OSV-S, 15 items) and basic social demographic data (T0a). Participants with an inconspicuous score receive a psychoeducational text with preventive media use strategies. Participation in the OMPRIS intervention can still take place if desired. Participants with at least harmful use (OSV-S score above 7 points) of the internet will be directed to create a user account. The creation of a user account only requires the assignment of a self-selected pseudonym and an e-mail address.
2. Afterwards, the participants book a first appointment for participation education. This webcam-based individual interview serves to inform the participants about the purpose of the study and to present the study procedure in detail. The inclusion, discontinuation and exclusion criteria are recorded in a standardised manner before participation in the intervention in the course of the telemedical participation clarification interview, which is carried out by a trained psychologist via webcam. For this purpose, a structured clinical interview for the recording of mental disorders (MINI) is carried out within the framework of the participation clarification interview, which records the psychiatric diagnoses of the exclusion criteria.
3. After the participants have been informed verbally, an online-based, active consent to participate in the OMPRIS study takes place on the portal by the participant and, if necessary, separately by a parent (if under 18 years of age). The explicit consent of the participant or the parent/guardian to participate in the study is obtained via the study platform.
4. The biased-coin randomisation (intervention vs. waiting group), taking into account gender distribution and symptom severity, will take place afterwards.
5. Then the participant selects the appointment slots for the individual intervention and the appointment for the initial interview.
6. This is followed by the questionnaire survey (pre-measurement) (T0c).
7. Structured diagnostic interview: Internet-related disorder (AICA and MINI) for IG and WG, assessment GAF. Start of 4-week individual counselling; In the intervention group, a total of 9 webcam-based individual counselling sessions and optionally 1 social counselling session are offered. Table 2 lists the individual intervention foci with example interventions. The intervention is thematically manualised and should be as standardised as possible in all individual counselling sessions (exception: individual topics, social counselling).
8. After 2 weeks of the intervention/waiting period, participants (IG and WG) are asked to complete a few questionnaires on symptom severity and motivational attitude for follow-up (T1).
9. After completion of the intervention, the post-treatment data collection (T2) (post-measurement) will be conducted. As an allowance for completing the questionnaires, each participant will receive a fee of 20 Euros after data entry in order to keep the drop-out rate minimal. At the same time, the waiting control group starts with the OMPRIS intervention as in point 7) and participates in the post-treatment survey after completion.
10. The follow-up data collection takes place 6 weeks (T3a/b) and 6 months (T4) after the end of the OMPRIS intervention. Completion of the questionnaires at T3 is again remunerated with 15 euros each in order to reduce the drop-out rate. Compensation for completing T4 will be sought (5 euros). A reallocation of funds will be requested in this regard.

| Table 2: OMPRIS intervention examples. | |
| --- | --- |
| **Treatment direction** | **Intervention examples** |
| Motivational Interviewing | Client-centred, guided counselling approach with the aim of building intrinsic motivation to change behaviour. The concept was developed for counselling people with addiction problems and is widely used in addiction medicine, but also for other mental disorders (Lawrence et al., 2017). |
|  |  |
| Behavioural therapy elements | Education, establishment of a disorder model, cost/benefit considerations, behaviour diaries, exposure, own rules regarding media use, affect regulation (especially boredom and negative affect) |
|  |  |
| Addiction therapy elements | Relapse prevention, abstinence round, addiction triangle, peer group, problematic situations, "emergency" measures in case of addiction pressure |
|  |  |
| Everyday structure | Sleep hygiene, sleep-wake rhythm, eating structure, personal hygiene, social situations, basic needs |
|  |  |
| Social work | Help with applications, unemployment, social integration, debts, hobby search |

**4.7 Sample size calculation:** The sample size calculation was performed using a two-sided two-sample t-test at a significance level of 5% and to achieve a power of 80%. For the standard deviation, a value of sd = 3.92 was used, which corresponds to the observed standard deviation in the data of the comparable STICA study (psychotherapy study for Internet addicts). For a difference to be detected of δ =2 points in the OSV-S, which corresponds to about one third of the difference observed in the STICA study, a required number of cases of 62 participants per group results. Assuming a drop-out rate of 30%, 81 participants per group should be included in the study.

**4.8 Randomisation:**

Participants are randomly assigned to either the OMPRIS intervention or the waiting control group. The biased-coin randomisation, taking into account gender distribution, sort of internet use, and symptom severity, is computer-generated by the online-based environment. The intervention group takes place in the form of individual consultations. Since the intervention is conducted independent of location, randomisation does not take place on a centre-specific basis.

**4.9 Evaluation strategies and quality assurance**:

Recruitment is supported by the cooperation with the Fachverband Medienabhängigkeit e.V. as well as the the ZTG Centre for Telematics and Telemedicine Bochum (dissemination concepts online, usability). The number of participating study/provider centres ensures that a sufficient number of affected persons can participate in the study. The online-based, i.e. location-independent intervention can provide findings that are representative for Germany and transferable to standard care. The evaluation combines quantitative symptom-related and health economic methods and includes different effect sizes. For quality assurance purposes, a detailed study plan with procedures and defined areas of responsibility will be drawn up. Escalation rules are defined in the event of delays in the submission of data, and plausibility checks and analyses of drop-outs are carried out. Statistical advice, planning and analysis are carried out by the Department of Medical Informatics, Biometry and Epidemiology at the Ruhr University Bochum.

**4.10 Evaluation design and statistical analyses**:

1) Clinical outcome variables: The analyses of the primary and secondary endpoints are carried out by means of a covariance analysis on the differences of the respective scores between the time points T0c and T1. The variable of interest is the intervention group; in addition to the value of the score considered at T0c (baseline), the following variables are also included in the model as covariates: Type of Internet addiction, gender and presence of psychiatric comorbidity. The analysis is carried out in the intention-to-treat population. Missing values are to be replaced by means of imputation procedures. Since it can be assumed that these are not "missing-at-random", various sensitivity analyses with different replacement strategies, in particular also conservative replacements, are to be calculated in order to estimate the influence on the results. The details will be specified in the Statistical Analysis Plan. A two-sided significance level of 5% will be used for the analysis of the primary endpoint. In exploratory analyses, the influence of different predictors on treatment success will be investigated using linear and logistic regression models.

2) Health economic target variables: The *health economic evaluation is* carried out by the Chair of Medical Management at the University of Duisburg-Essen (Head: Prof. Dr. J. Wasem). Within the framework of this, a cost-effectiveness analysis is carried out with the aim of determining the cost-effectiveness of the intervention in comparison to not using the intervention. Methodologically, a survey of the resource consumption of the persons with Internet addiction and problematic use of the Internet included in the study is carried out in the intervention group and the waiting group, which serves as the control group. With regard to the evaluation of the costs, a generic questionnaire for determining resource consumption in the health care system is adapted to the specific needs in the indication "Internet addiction". Retrospective data on outpatient doctor contacts, medication, inpatient stays and rehabilitation measures will be asked. With regard to indirect costs due to productivity losses, questions on employment status are added to the survey of socio-demographic data and information on incapacity for work and partial or complete reduction in earning capacity is determined in the health economic questionnaire. According to the quantity structure of resource consumption generated in this way, a price structure (taking into account the general principles of health economic evaluation and including published valuation rates) is developed (von der Schulenburg 2007, Bock et al. 2015). In addition, the resource consumption by the intervention is also collected and the intervention costs are determined analogously to the previously described procedure. As an effect parameter, the severity of the Internet addiction among the study participants affected by Internet addiction is used. As a result, the cost-effectiveness of the intervention is determined in comparison to not using the intervention.

**4.11 Data collection:**

Primary data (Table 1) will be collected through a) self-assessment instruments to collect different variables and b) clinical, standardised diagnostic interviews (third-party assessment by practitioner). Existing data sets or primary data (e.g. health insurance data) are not used. All self-assessment data are collected through online-based questionnaires. The data is encrypted and stored on a secure server. A privacy policy has been prepared accordingly and is attached separately to this study protocol.

**4.12 E-health interface:** The e-health solution used for use and participation in this project is freely accessible via the internet in the form of a homepage. No software needs to be loaded. The IT environment used is based on software from a video service provider. The participants create a user account. All data is stored on a secure server.

5 Risk-benefit assessment

**5.1 Risks & burdens associated with study participation**

Possible risks in the realisation of the project are mainly that (i) personal data are misused and/or (ii) participants get into psychological crises and need help.

Re (i): Data protection compliance is of great importance for the project. All partners have great experience with the implementation of data protection concepts in everyday clinical practice and clinical studies. All data protection concepts are agreed with the responsible ethics committees and data protection officers, so that there should be no risk of data collected being misused. In addition, the data are anonymised and thus it is not possible to draw conclusions about the person. Re (ii): The treatment centres involved are (partly university) specialist clinics in the field of psychosomatic medicine and psychotherapy and therefore offer a suitable infrastructure for the treatment of mental crises. In case of emergency, the participants' clear names and contact details are stored separately from the results of the questionnaires. The participants are informed about this procedure and must consent to it.

#### 5.2 Individual benefits associated with study participation

The OMPRIS care research project presented here is an innovative, new care strategy for those affected by harmful use or dependence on computer games and Internet addiction. It offers early and low-threshold treatment in the "addiction space" of the internet. This complements conventional treatments, primarily for psychological comorbidities (especially depression and anxiety disorders), and makes them more efficient. Many Internet users with problematic use of the Internet can be offered a (secondary) preventive intervention at an early stage to counteract a further development of addiction and the associated need for therapy. Ultimately, the OMPRIS project offers a low-threshold and location-independent intervention that can close the currently prevailing gap in the specific treatment of people with Internet addiction. Those affected can also be guided to specific local services within the framework of the OMPRIS study.

**Participant orientation:** The OMPRIS project is participant-oriented. Access is free for all internet users via a homepage. Through the freely accessible screening, those affected can already be given a first assessment of their internet use. Further steps are taken after the creation of a user account and consent to participate in the study. A structured diagnostic process is offered in an uncomplicated way, independent of location (because it is online), with the aim of offering the person affected participation in OMPRIS according to his/her disorder. The counsellors' basic approach and the way in which they conduct discussions is always oriented towards motivating the person by means of the established Motivational Interviewing. Nevertheless, the person concerned has to be very active in the programme (participation). The early webcam-based relationship between the person concerned and the counsellor promotes bonding and familiarity, which has a positive effect on the adherence of the person concerned. The exchange among like-minded people is also an important factor that contributes to the success of motivational programmes.

**Organisational, procedural innovation, care processes:** The online-based service eliminates travel distances and external barriers that can prevent intervention. The great scheduling/time variability of the online offer allows maximum flexibility for the person concerned and thus lowers external barriers. Thanks to the expertise of the study centres and the networking with the Fachverband Medienabhängigkeit e.V., those affected can be offered the best possible current procedural infrastructure for the treatment of internet addiction.

**Optimisation of the medical outcome:** The early, location-independent intervention offers within the framework of the OMPRIS project are intended to improve problematic media use behaviour, motivation to change, quality of life and social functioning in a (secondary) preventive way. In addition, specific intervention strategies of the OMPRIS project are to reduce addiction symptoms, depressive and anxiety symptoms in order to prevent chronification processes at an early stage. Those affected by internet addiction can be offered support for referral to analogue, disorder-oriented therapeutic help if they are motivated to change.

**Quality and cost-effectiveness: The** evaluation concept will be used to examine clinical outcome variables, health economic issues and the participants' satisfaction with the project.

**5.3 Statement on medical justifiability**

In the context of the current professional policy decisions to open up telemedical services also in the field of treatment of mental disorders, the establishment of the new OMPRIS service is to be supported. The project also has a strong preventive character. OMPRIS does not claim to replace necessary medical or psychotherapeutic treatment, but offers an additional service in a medically underserved (because it is so new) sector. From a medical point of view, the project is therefore justifiable.

6 Data management and data protection

A separate description of the data protection concept according to the current General Data Protection Regulation 2018 (GDPR) is attached to the study protocol, which explains all relevant points. The information provided here is therefore to be understood as an overview.

The legal basis of this Innovation Fund project is a consortium agreement based on §§ 92a and 92b SGB V between the consortium partners, which regulates the uniform project implementation and rights and obligations between the contractual partners. In addition, the usual ethical and scientific standards apply. **Protection of participants:** Participation is voluntary and subject to the submission of a participation and data protection declaration. The project is submitted to the relevant ethics committees for assessment before it is launched. **Legal aspects:** Since the OMPRIS project is primarily a preventive online-based counselling service, the "ban on remote treatment" according to § 7 para. 4 MBO - Ä is not relevant for the planned procedure. No medical treatment or psychotherapy in the strict sense is carried out. **Data protection:** Participation requires the consent of the persons concerned for data storage/analysis. For the scientific evaluation, all participant-related data will be linked by assigning a self-selected alias name. The consortium partners and subcontractors have extensive experience with regard to the necessary measures for participant and data protection. **Access to secondary data:** No secondary data, e.g. from health insurance companies, will be used. **Overarching assessment of the risks and benefits of the project: Overall,** no additional medical risks arise for the study participants as a result of the project. Access to a doctor and the medical assessment of changes and in particular a worsening of symptoms are guaranteed at all times.

7 Signatures


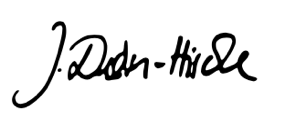


____Bochum,30.01.2020_____________________________________________________________ _

Place, date *Signature*

Jan Dieris-Hirche, MD

**(Overall) project management/consortium management**

8 References

American Psychiatric Association APA (2015): Diagnostic and Statistical manual of mental disorder (5th ed.), DSM-5. Washington DC: APA.

Bassler, M., Potratz, B. & Krauthauser, H. (1995). Luborsky's Helping Alliance Questionnaire (HAQ). Psychotherapist, 40, 23-32.

Bishop G et al. (2017): Treatment manual internet-related disorders. University of Lübeck.

Bock JO, Brettschneider C, Seidl H, Bowles D, Holle R, Greiner W, et al. (2015): Calculation of standardised unit costs from a societal perspective for health economic evaluation. Gesundheitswesen. 2015;77(1):53-61.

Brunborg GS, Mentzoni RA, Frøyland LR (2014): Is video gaming, or video game addiction, associated with depression, academic achievement, heavy episodic drinking, or conduct problems? J. Behav. Addict. 2014; 3:27-32.

DAK Health (2015): Abschlussbericht DAK Studie Internetsucht im Kinderzimmer. Retrieved online 7.2.2018: https://www.dak.de/dak/download/praesentation-dak-studie-1730008.pdf

DAK Gesundheit (2018): DAK - Digitalisierungsreport 2018. This is how doctors think about e-health solutions. Retrieved online 7.2.2018: https://www.dak.de/dak/download/dak-digitalisierungsreport-2018-1959528.pdf

de Zwaan M, Herpertz S, Zipfel S et al. (2017): Effects of Internet-Based Guided Self-help vs Individual Face-to-Face Treatment on Full or Subsyndromal Binge Eating Disorder in Overweight or Obese Patients: The INTERBED Randomized Clinical Trial. JAMA Psychiatry. 2017;74(10):987-995.

The Drug Commissioner of the Federal Republic of Germany, Federal Ministry of Health (2017): Drugs and Addiction Report 2017 of the Federal Government. Retrieved online 7.2.2018: https://www.drogenbeauftragte.de

Graf von der Schulenburg J-M, Greiner W, Jost F et al. (2007): German recommendations on health economic evaluation - third and updated version of the Hanover Consensus. Health Economics & Quality Management 2007; 12: 285-290.

Initiative 21e.V. (2018): Final report D21-Digital-Index 2017 / 2018, a study by Initiative D21 e.V. Online. abgerufen am 7.2.2018: http://initiatived21.de/app/uploads/2018/01/d21-digital-index_2017_2018.pdf.

Jäger S, Müller KW, Ruckes C et al. (2012): Effects of a manualized short-term treatment of internet and computer game addiction (STICA): study protocol for a randomized controlled trial.Trials. 2012 Apr 27;13:43.

Lawrence P, Fulbrook P, Somerset S, Schulz P (2017): Motivational interviewing to enhance treatment attendance in mental health settings: A systematic review and meta-analysis. J Psychiatr Ment Health Nurs. 2017 Nov;24(9-10):699-718. doi: 10.1111/jpm.12420. Epub 2017 Sep 14.

Lambert MJ (2013): Outcome in Psychotherapy: The Past and Important Advances. Psychotherapy. 2013, Vol. 50, No. 1, 42-51.

Lemmens JS, Valkenburg PM, Peter J (2011): Psychosocial causes and consequences of pathological gaming. Comput. Hum. Behav. 2011; 27: 144-152.

Lee, June-Young, et al. (2014): The Difference in Comorbidities and Behavioral Aspects between Internet Abuse and Internet Dependence in Korean Male Adolescents. Psychiatry investigationon 11.4 (2014): 387-393.

Löwe B, Spitzer RL, Zipfel S, Herzog R (2002): *Patient Health Questionnaire (PHQ D). Complete version and short form.* Test folder with manual, questionnaires, templates. 2nd edition. Pfizer, Karlsruhe.

Mihara S, Higuchi S (2017): Cross-sectional and longitudinal epidemiological studies of Internet gaming disorder: A systematic review of the literature. Clin Neurosci. 2017 Jul;71(7):425-444.

Müller K & Wölfling K (2017): Structured clinical interview for Internet-related disorder AICA-SKI:IBS. Available at: http://www.fv-medienabhaengigkeit.de/fileadmin/images/Dateien/AICA-SKI_IBS/Quickreader_AICA-SKI_IBS.pdf.

Orth, B. (2017): The drug affinity of adolescents in the Federal Republic of Germany 2015. Subvolume computer games and internet. BZgA research report. Cologne: Federal Centre for Health Education. Retrieved online 7.2.2018: <https://www.drogenbeauftragte.de>

Rammstedt, B., Kemper, C. J., Klein, M. C., Beierlein, C. & Kovaleva, A., (2013). A Short Scale for Assessing the Big Five Dimensions of Personality - 10 Item Big Five Inventory (BFI-10). [A Short Scale for Assessing the Five Dimensions of Personality - 10 Item Big Five Inventory (BFI-10)]. Big Five Inventory (BFI-10). *methods, data, analyses,* 7(2), 233-249.

Rumpf JH et al. (2011): Prevalence of internet addiction PINTA. Report to the Federal Ministry of Health. Retrieved online 7.2.2018: <https://www.bundesgesundheitsministerium.de>

Schöttke, H., Lange, J., Imholz, M. & Wiedl, K. H. (2011). Development of a screening procedure for the diagnosis of personality disorders: The Personality Disorder Screening - Short Form (PSS-K). *Behaviour Therapy*, *21*(3), 154-161.

Sheehan D et al. (2010): M.I.N.I. Mini-International Neuropsychiatric Interview 6.0.0 DSM-IV. Tampa: University of South Florida.

The EuroQol Group (1990). EuroQol-a new facility for the measurement of health-related quality of life. Health Policy 16(3):199-208.

Weinstein AM (2017): An Update Overview on Brain Imaging Studies of Internet Gaming Disorder. Psychiatry. 2017 Sep 29;8:185. doi: 10.3389/fpsyt.2017.00185. eCollection 2017.

Wölfling K et al (2010): Diagnostic test procedures: Scale for online addictive behaviour in adults. In: Mücken et al. (eds.). Prevention, diagnostics and therapy of computer game addiction. Lengerich: Pabst.

Wölfling K et al. (2013): Computer game and internet addiction: a cognitive-behavioural treatment manual. Stuttgart: Kohlhammer.

World Health Organisation WHO (2018). The ICD-11 classification of mental and behavioural disorders. ICD 11 Beta Draft online, Retrieved 7.2.2018 online: https://icd.who.int/dev11/l-m/en
