# Supplementary material for: Efficacy of a short-term webcam-based telemedicine treatment of internet use disorders (OMPRIS): a multicentre, prospective, single-blind, randomised, clinical trial
Source: eClinicalMedicine. 2023 Sep 14;64:102216. doi: 10.1016/j.eclinm.2023.102216 (PMC10514435; doi:10.1016/j.eclinm.2023.102216)
Supplement: Statistical Analysis Plan [file mmc3.docx]

**Statistical Analysis Plan**

**Study acronym :**

**Title of the study:**

**Based on protocol version and date:**

**Statistics:**

**Principal Investigator:**

**OMPRIS**

Online-based motivational programme to reduce problematic media use and promote treatment motivation in people with gaming disorder and internet addiction: a multicentre, prospective, randomised controlled trial (RCT) with a wait-list control group

This document has been written based on the information contained in the published study protocol (accepted 22 June 2021).

Prof. Dr. Nina Timmesfeld

Marianne Tokic

Jale Basten

Dr. med. Jan Dieris-Hirche

Prof. Dr. med. Stephan Herpertz

**Verified by:**


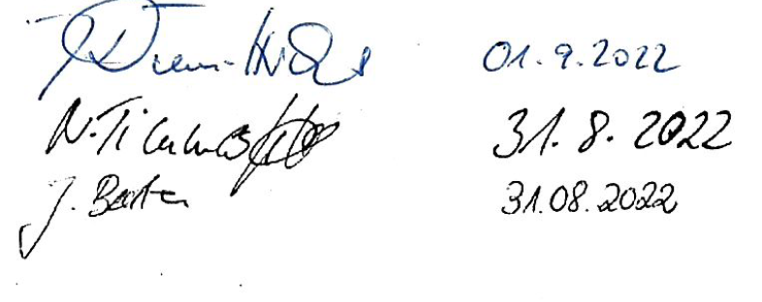
**Name: Unterschrift: Datum:**

1) Dr. med. Jan Dieris-Hirche

2) Prof. Dr. Nina Timmesfeld

3) Jale Basten

**SAP Version:** final

Content

[0 Introduction 5](#_Toc129968460)

[0.1 Preface 5](#_Toc129968461)

[0.2 Background 5](#_Toc129968462)

[0.3 Research questions of the study 6](#_Toc129968463)

[0.4 Project Management 6](#_Toc129968464)

[1 Study Design and Procedure 7](#_Toc129968465)

[1.1 Study design 7](#_Toc129968466)

[1.2 Randomisation 9](#_Toc129968467)

[1.3 Blinding 9](#_Toc129968468)

[1.4 Case number calculation and clinical case number estimation 10](#_Toc129968469)

[1.5 Study population 10](#_Toc129968470)

[2 Outcomes 12](#_Toc129968471)

[2.1 Objective variables and survey instruments used 13](#_Toc129968472)

[2.2 Measuring times 15](#_Toc129968473)

[3 General consideration 17](#_Toc129968474)

[3.1 Timing of the analyses 17](#_Toc129968475)

[3.2 Analysis population 17](#_Toc129968476)

[3.2.1 Full Analysis Set (FAS) 17](#_Toc129968477)

[3.2.2 Modified ITT data set 17](#_Toc129968478)

[3.2.3 Per-Protocol (PP) data set 17](#_Toc129968479)

[3.3 Methods of analysis 17](#_Toc129968480)

[3.4 Covariates 17](#_Toc129968481)

[3.5 Missing Data 17](#_Toc129968482)

[4 Summary of the study data 18](#_Toc129968483)

[4.1 Description of the patient flow 18](#_Toc129968484)

[4.2 Demographic and baseline variables 19](#_Toc129968485)

[5 Efficacy analyses 21](#_Toc129968486)

[5.1 (Modified) ITT Analyses 21](#_Toc129968487)

[5.2. Per-Protocol Analysis 23](#_Toc129968488)

[5.3 Sensitivity analyses 23](#_Toc129968489)

[5.4 Subgroup analyses 23](#_Toc129968490)

[5.5 Moderator analyses 23](#_Toc129968491)

List of abbreviations

AICA-S Assessment of Internet and Computer Game Addiction Scale

AICA-SKI: IBS Assessment of Internet and Computer Game Addiction – Structured Clinical Interview

BFI-10 Big Five Inventory 10-item version

CIUS Compulsive Internet Use Scale

DSM-5 Diagnostic and Statistical Manual of Mental Disorders Fifth Edition

EP Endpoint

EQ-5D-5L EuroQoL Standardised Measure of Health-related Quality of Life–5 Dimensions, 5-Level version

GAD-7 Generalized Anxiety Disorder Screener 7-item version

GSE General Self-Efficacy Scale

HAQ Helping Alliance Questionnaire

IG Intervention group

IGD internet gaming disorder

iSocrates Stages of Change Readiness and Treatment Eagerness Scale for
 Internet–Addiction

ICH International Conference on Harmonization

ITT Intention-to-treat

IUD internet use disorder

L-1 General Life Satisfaction 1-item version

MINI Mini-International Neuropsychiatric Interview

PHQ-9 Patient Health Questionnaire 9-item version

PP Per-Protocol

RCT randomized controlled trial

SAP Statistical analysis plan

STICA Short-term treatment of internet and computer game addiction

SUS System Usability Scale

WHO World Health Organization

WCG Waiting control group

# 0 Introduction

## 0.1 Preface

The purpose of the statistical analysis plan (SAP) is to ensure the credibility of the study results by defining the statistical analysis procedures for the study data before the database is locked and unblinded. To avoid bias in the results and selective reporting, a detailed SAP is presented to avoid post-hoc decisions that could influence the interpretation of the results of the statistical analyses of the final data.

This SAP is a technical extension of the clinical trial protocol published in BMJ Open^[[1]](#footnote-1)^, and follows the principles of the International Conference on Harmonization (ICH) guidelines E3, E6 and E9.

## 0.2 Background

In 2019, around 90% of all German households had access to the World Wide Web. Families with at least one child have almost 100% internet coverage.^[[2]](#footnote-2)^ A recent representative study conducted with German adolescents reported increased time spent with internet applications, with a particular increase in 2020 due to the COVID-19 pandemic. The average time spent with video games was 139 minutes on weekdays and 193 minutes on weekends.^[[3]](#footnote-3)^ In addition, there are other studies from other countries that indicate an increase in gaming behaviour (e.g. hours played) among students and adolescents, especially due to the COVID-19 pandemic in 2020.^[[4]](#footnote-4),^^[[5]](#footnote-5),^^[[6]](#footnote-6)^

In May 2019, the WHO will classify internet gaming disorder (IGD) as a mental disorder in the upcoming International Classification of Diseases (11th revision). However, those affected by IGD or internet use disorders (IUDs) often do not receive appropriate therapy because they lack insight and motivation or do not have access to suitable local treatment options. The still incomplete care situation for the specific treatment of people with computer game and internet addiction as well as the addiction-specific motivation problems currently lead to an underuse of those affected, who usually come into treatment very late - often under pressure from the social system. This leads to chronic courses of the disease, in which there can be clear social functional deficits (loss of education or job, social isolation, neglect).

If those affected seek psychotherapeutic treatment, it is usually the psychological comorbidities (depression, ADHD, anxiety disorders) that are treated; internet or computer game addiction is usually not treated adequately due to a lack of disorder-oriented therapy approaches. In addition, the harmful use of the internet, but also internet addiction, is still played down socially and therapeutically. A possible early, (secondary) preventive intervention is thus often considered too late. 

The above derivation results in the following project goals, which are to be worked on within the framework of the OMPRIS study:

1) Creation of a low-threshold and early applicable online-based guided, structured, manualised counselling offer to "pick up" affected and endangered people in the "addiction space" internet.

2) Targeted and efficient promotion of motivation to change behaviour through a compact, location-independent (because online-based), guided, structured, manualised counselling offer to reduce media addiction symptoms and secondary prevention of further addiction problems.

3) If internet addiction is present: Targeted support for referral to internet addiction-oriented support services (e.g. specialised treatment centres) in order to improve ongoing treatment of comorbid disorders and thus make treatment more efficient.

## 0.3 Research questions of the study

The primary aim of the study is to examine whether participation in the study leads to a reduction in media addiction symptoms, i.e. problematic media use behaviour. In addition, further secondary analyses will examine the increase in motivation to change regarding problematic media use, the increase in quality of life, the reduction in depressiveness and anxiety, the increase in social/global psychological functioning and the increase in self-efficacy.

Descriptively, the therapeutic relationship, placement rate in analogue treatment services, personality traits and satisfaction with the system will be presented. Exploratively, it will be examined whether there are possible predictors (e.g. person-related characteristics) for successful participation in OMPRIS and the placement rate in analogue treatment offers will be surveyed.

## 0.4 Project Management

Consortium leader, project management and applicants: Dr. med. Jan Dieris-Hirche, Tel.: +49-234-5077-3135, Fax: +40-234-5077-3759. Email: jan.dieris-hirche@rub.de

Consortium leader, director of the clinic: Prof. Dr. med. Stephan Herpertz, Tel.: Tel.: +49-234-5077-3100, Fax: +49-234-5077-3111. Email: stephan.herpertz@rub.de.

Both work at the LWL University Hospital of the Ruhr University Bochum, Department of Psychosomatic Medicine and Psychotherapy, Alexandrinenstraße 1-3, 44791 Bochum, Germany.

# 1 Study Design and Procedure

## 1.1 Study design

The multicentre, prospective, single-blind OMPRIS study is planned as a randomised controlled trial (RCT) in a waiting group control design (Figure 1). All participants in the study will be randomly assigned to either the immediate intervention group (IG) or the waiting control group (WCG, 4-6 weeks). The primary endpoint will be measured continuously during the 4 weeks of the intervention (measurement points: T0 baseline, T1 mid-intervention and T2 post-intervention). During these 4 weeks, subjects will be offered up to eight webcam-based psychological treatments and one or two social support sessions. In addition, an in-depth diagnostic webcam session will be offered 1 week before and 1 week after the intervention. Two follow-up surveys, one 6 weeks after the end of the intervention (T3) and 6 months after randomisation (T4) will take place. The study is funded by the Innovation Fund of the Federal Joint Committee and is therefore primarily a health research study designed to investigate an innovative form of telemedical eHealth care.

Figure 1: Flow chart of the survey time points: Eligibility assessment (T0a), baseline (T0), mid-intervention (T1; after 2 weeks), post-intervention (T2; after 4 weeks) and two follow-ups (T3: 6 weeks after intervention, T4: 6 months after randomisation).

## 1.2 Randomisation

With sequential balancing randomisation according to Borm et al^[[7]](#footnote-7)^, prognostically relevant factors are balanced in sequential order. In this method, each factor is treated sequentially, and when new subjects enter the OMPRIS intervention, they are assigned to either IG or WCG, depending on which assignment leads to better equilibrium of the first factor in the arms. For example, if there is equilibrium on the first factor, then the subject is assigned to the group that results in improved equilibrium on the second factor. When all factors are balanced according to the predefined imbalance levels, the new subject is randomly assigned. Four factors have relevant prognostic value, each of which is categorised into three classes based on data from a previous study^[[8]](#footnote-8),^^[[9]](#footnote-9)^, and the AICA-S questionnaire^[[10]](#footnote-10),^^[[11]](#footnote-11)^: (1) gender (female, male, diverse); (2) severity of Internet-related addiction symptoms (AICA-S <7, 7-13; >13); (3) age (16-17, 18-30, >30 years); and (4) type of Internet addiction (online gaming, pornography/cybersex, all other genres). The Department of Medical Informatics, Biometry and Epidemiology in Bochum, which is not involved in the OMPRIS enrolment or evaluation, is responsible for determining the imbalance levels and for conducting the randomisation. As the intervention is conducted location-independently, randomisation does not take place on a centre-specific basis.

## 1.3 Blinding

The study will be conducted in a single-blinded design. Participants will be informed after the first introductory session that they have been randomly assigned to either the IG (who will start the intervention immediately) or the WCG (who will start the intervention after a four-week delay).

In addition, staff delivering the OMPRIS intervention are not informed about the groups assigned to participants. Outcome measurement is software-based (self-report). Participants will receive a short, automatically generated personal feedback report by email after their last session of the OMPRIS intervention. The study database will remain blinded during data preparation (no group variable) and during the evaluation (no knowledge of group assignment behind the labels).

## 1.4 Sample size

The sample size was calculated using a two-sided two-sample t-test at a significance level of 5% and to achieve a power of 80%. A recent RCT treatment study (STICA study for the short-term treatment of internet and computer game addiction) showed an effect size of d=1.19 for the effect of analogue cognitive behavioural therapy on reducing internet-related use disorder symptoms (SD=3.92) using the same outcome measure AICA-S. ^[[12]](#footnote-12)^ For a conservative effect size of d=0.51 (corresponding to about 43% of the STICA study) and a difference to be detected of δ =2 points in the AICA-S, this results in a required a sample size of 62 patients per group. Assuming a drop-out rate of 30%, 81 patients per group should be included in the study.

## 1.5 Study population

The primary target population of the OMPRIS study are those affected by problematic or pathological use of computer games, other specific types of internet use (internet pornography, social networking sites, etc.) or the internet in general. However, in order to promote the preventive approach of the intervention, subjects who have achieved an unremarkable score in the screening are also admitted on request.

In order to enable the lowest possible threshold for participation, broad **inclusion criteria** were chosen:
1) Possibility of regular use of an internet connection with webcam and/or at least microphone as well as an email address.

2) Age from 16 years (under 18 years with the consent of at least one parent in the form of a written consent and a short webcam-based confirmation).

3) Sufficient understanding and speaking of the German language

4) Informed consent to terminate pseudonymisation in an emergency (i.e. in case of concrete suicidal tendencies).

**Exclusion criteria** were defined as:

1.) Acute or past psychotic symptoms in the last 6 months (acute delusions, hallucinations, massive thought disorders).

2) Acute suicidal tendencies or self-endangerment

3.) Severe intelligence impairment that prevents participation

4) Substance dependence in the foreground

5) Age under 16 years or lack of consent of the legal guardian in the case of minors.

6) Lack of German language skills

7) Lack of regular internet access with webcam and lack of email address.

8) Presence of known previous somatic illnesses which themselves or their treatment with hormone-influencing medication could be associated with an impulse control disorder (e.g. Parkinson's disease with dopaminergic medication).

9.) Currently already undergoing psychiatric or psychotherapeutic treatment due to media addiction.

10) Consumption of illegal content (added subsequently for reasons given; not mentioned in the study protocol as an exclusion criterion).

# 2 Outcomes

Primary endpoint

Participation in OMPRIS leads to a reduction of media addiction symptoms, i.e. problematic media use behaviour, in the participants. The primary outcome measure is the Adult Online Addiction Scale (AICA-S, Wölfling et al., 2010) at the end of the intervention (T2). If the primary endpoint is not available at T2, it is replaced by the AICA-S at T1 (2 weeks after the start of the intervention, i.e. midpoint of the intervention), if available. If the AICA-S is not available at either T1 or T2, imputation is used (see 3.5 Missing data).

Secondary endpoints

1) Participation in OMPRIS leads to an increase in participants' motivation to change their problematic media use as measured by the Stages of Change Readiness and Treatment Eagerness Scale (iSOCRATES-D, Miller & Tonigan, 1996).

2) Participation in OMPRIS leads to an increase in the participants' quality of life. Health-related quality of life is measured with the EQ5D (EuroQol Group, 1990; to be specified in a separate SAP for the health economic data). General life satisfaction is measured with the single-item scale L-1 (Beierlein, Kovaleva & Lázló, 2014).

3) Participation in OMPRIS leads to a reduction in depression and anxiety in participants, measured with the relevant questions (PHQ-9 and GAD-7) of the Patient Health Questionnaire (PHQ-D, Löwe et al., 2002).

4) Participation in OMPRIS leads to an increase in participants' social/global psychological functioning as measured by the Global Assessment of Functioning (GAF, American Psychiatric Association, 1989).

5) Participation in OMPRIS leads to an increased perception of self-efficacy among participants, measured with the General Self-Efficacy Scale (GSE, German translation SWE, Schwarzer et al., 1995).

6) Participation in OMPRIS leads to a reduction of internet-related disorders in the participants, measured with the Compulsive Internet Use Scale (CIUS, Meerkerk et al., 2009).

The secondary endpoint is the respective target parameter after completion of the intervention (T2). If the measured value is not available at T2, it is replaced by the measured value at T1, as with the primary endpoint. If neither the measured value at T1 nor at T2 is available, the observations of the persons are excluded (modified ITT).

Further descriptive measurements

Descriptively, the following results are presented: Therapeutic relationship from patient and therapist perspective, placement rate, personality traits, subjectively perceived symptom severity, psychiatric comorbidities, satisfaction with technical implementation.

## 2.1 Objective variables and survey instruments used

Table 1: Objective variables and survey instruments used

| **Instrument** | **Construct** | **Items** |
| --- | --- | --- |
| **Primary outcome variable: Reduction of internet-related addiction symptoms** | | |
| AICA Addiction Criteria DSM-5 (Müller & Wölfling, 2017) | - Scale for online addictive behaviour in adults  - Covers all relevant addiction criteria oriented to the DSM-5  - Questions on a five-point Likert scale from 0 = "never" to 4 = "very often". | 15 |
| **Secondary outcome variables** | | |
| Extent of improvement of a motivation for change | | |
| iSOCRATES-D (Miller & Tonigan, 1996) | - Internet-related version of the Stages of Change Readiness and Treatment Eagerness Scale - Specially developed German version of the SOCRATES scale established in addiction medicine (Miller & Tonigan, 1996), adapted for Internet addiction. - Questions on a five-point Likert scale from 1 = "do not agree" to 5 = "completely agree". | 19 |
| Increase in quality of life | | |
| L-1 (Beierlein, Kovaleva & László, 2014) | - Scale includes only one item "How satisfied are you, all things considered, with your life at present?", answered on an 11-point scale from "not at all satisfied" (0) to "completely satisfied" (10). - Reliability was tested by test-retest reliability, which was given as rtt=0.67. | 1 |
| Reduction of clinical depressiveness and anxiety. | | |
| PHQ-9 (Spitzer et al., 1999) | - Self-report version of the Primary Care Evaluation of Mental Disorders (PRIME-MD) screening instrument for common mental disorders.  - Each of the nine DSM-IV criteria is rated on a four-point Likert scale from 0 = "not at all" to 3 = "almost every day".  - Excellent internal consistency (α=0.83-0.92) | 9 |
| GAD-7 (Spitzer et al., 2006) | - Measure assessing general anxiety symptoms according to DSM-IV criteria on a four-point Likert scale.  - Excellent internal consistency (α=0.89) | 7 |
| Increase in social/global mental functioning. | | |
| GAF (American Psychiatric Association, 1989) | - Scale for the psychosocial functioning level of a patient, independent of their cultural background and the severity of their symptoms.  - GAF scale is divided into 10 levels of functioning with 10 points each  - ranges from 100 (highest level of functioning) to 1 (lowest level of functioning) | 1 |
| Increased perception of self-efficacy | | |
| GSE (Schwarzer & Jerusalem, 1999) | - General self-efficacy expectation scale  - four-point scale from "not true" to "true exactly".  - Measures optimistic competence expectation, i.e. confidence in mastering a difficult situation, with success being attributed to one's own competence | 10 |
| Reduction of internet-related problems | | |
| CIUS (Meerkerk et al., 2009) | - Compulsive Internet Use Scale  - 5-point Likert scale  - Measures symptoms of internet-related disorders  - Has shown good internal consistency (α=0.89) | 14 |
| **Descriptively collected further parameters** | | |
| AICA-SKI:IBS (Müller & Wölfling, 2017) | - Elicitation of symptoms of Internet-related disorder evaluated by the Structured Clinical Interview on Internet-related Disorders.  - Interview is aligned with the DSM-5 criteria for Internet Gaming Disorder and provides a guided exploration of the major symptoms of Internet-related addiction  - was developed and validated on a sample of 500 outpatients | 6 |
| M.I.N.I. (Sheehan et al., 1998) | - Short structured diagnostic interview developed for DSM-5 and ICD-10 psychiatric disorders.  - with a completion time of about 15-20 minutes, it was developed to meet the need for a short but accurate structured psychiatric interview for multicentre clinical trials and epidemiological studies | 19 |
| Helping Alliance Questionnaire (HAQ; Bassler & Nübling, 1995) | - a highly relevant instrument for assessing the therapeutic alliance - can be used both as a patient version (HAQ-P) and, in a slightly modified form, as a therapist version (HAQ-T) - all items are rated on a 6-point Likert scale from "strongly agree" to "strongly disagree". - consists of two factors: "satisfaction with the therapy outcome" and "relationship with the therapist - Cronbach's alpha of the two scales was reported to be good (α=0.75-0.89 for the HAQ-P and α=0.63-0.85 for the HAQ-T) | 11 |
| Big Five Inventory (BFI-10; Rammstedt & John, 2007) | - Self-report with 10 items for the assessment of the Big Five personality traits - has five subscales with two bidirectional items for each of the personality factors - the 10 items are rated on a 5-point Likert scale where respondents can choose between answers ranging from "strongly disagree" to "strongly agree". - reliability was tested using test-retest reliability, which was found to be good (rtt=0.58-0.84) | 10 |
| Referral to other organisations | - Referral to other organisations and further treatment is assessed using three self-developed items | 3 |
| System Usability Scale (SUS; Brooke, 1996) | - Reliable tool for measuring usability across a wide range of services, including software, websites and applications.  - Consists of 10 items on a 5-point Likert scale from 'strongly agree' to 'strongly disagree'. | 10 |

Legende: AICA-S, Assessment of Internet and Computer Game Addiction Scale; iSOCRATES, Stages of Change Readiness and Treatment Eagerness Scale for Internet–Addiction; EQ-5D-5L, EuroQoL Standardised Measure of Health-related Quality of Life–5 Dimensions, 5-Level version; L-1, General Life Satisfaction 1-item version; PHQ-9, Patient Health Questionnaire 9-item version; SUS, System Usability Scale; SWE, Self-Efficacy Scale; GAD-7, Generalized Anxiety Disorder Screener 7-item version; MINI, Mini- International Neuropsychiatric Interview; GAF, Global Assessment of Functioning; CIUS, Compulsive Internet Use Scale; SWE, Self-Efficacy Scale; AICA-SKI:IBS, Assessment of Internet and Computer Game Addiction-Structured Clinical Interview.

## 2.2 Measuring times

Table 2: Questionnaires and measurement times

|  | **T0a**  Eligibility | **T0**  Baseline | **Each session** | **T1**  Mid-intervention | **T2**  Post-intervention | **T3 and T4**  Follow-up | |
| --- | --- | --- | --- | --- | --- | --- | --- |
| Expected point of data collection | 1 week |  |  | After 2 weeks | After 4 weeks | 6 weeks after T2 | 6 month after randomisation |
| Consent | x |  |  |  |  |  |  |
| Lifestyle Parameter |  | x |  |  | x | x | x |
| Treatment information |  | x |  |  |  |  |  |
| Satisfaction |  |  | x |  | x |  |  |
| Mood |  |  | x |  |  |  |  |
| AICA-SKI:IBS |  | x |  |  | x |  |  |
| **Group comparison** | | | | | | | |
| AICA-S | x | x |  | x | x | x | x |
| iSOCRATES |  | x |  | x | x | x | x |
| L-1 |  | x |  |  | x | x | x |
| PHQ-9 |  | x |  |  | x | x | x |
| GAD-7 |  | x |  |  | x | x | x |
| GAF |  | x |  |  | x |  |  |
| CIUS |  | x |  | x | x | x | x |
| GSE |  | x |  |  | x | x | x |
| **Descriptive evaluation (only available in intervention group)** | | | | | | | |
| HAQ |  |  |  | x | x |  |  |
| SUS |  |  |  |  | x |  |  |
| Referral rate |  |  |  |  |  | x | x |
| **Basic characteristics (Table 1)** | | | | | | | |
| Demographics | x |  |  |  |  |  |  |
| MINI |  | x |  |  |  |  |  |
| BFI-10 |  | x |  |  |  |  |  |
| **Health economics*** | | | | | | | |
| Resource use |  | x |  |  | x | x | x |
| EQ-5D-5L |  | x |  |  | x | x | x |

*Analyses on health economics are specified in a separate SAP.

# 3 General consideration

## 3.1 Timing of the analyses

The first subject was enrolled in the OMPRIS study on 1 September 2020. The recruitment of participants was completed in February 2022 and the follow-up data collection (T4) of the last subjects is expected by September 2022. After data processing, the database will be closed and the analysis and publication of the results is planned by the end of October 2022.

## 3.2 Analysis population

### 3.2.1 Full Analysis Set (FAS)

The analysis of the primary endpoint will be conducted as an intention-to-treat (ITT) analysis, i.e. all randomised subjects (n = 180) will be included in the analysis, regardless of early termination from the OMPRIS programme or outcome measurement.

### 3.2.2 Modified ITT data set

The secondary endpoints are evaluated in modified ITT populations. These include all randomised subjects who have the respective secondary endpoint at T0 and at T1 or T2.

### 3.2.3 Per-Protocol (PP) data set

The PP population consists of all participants who did not deviate significantly from the protocol. It was defined as subjects who participated in at least two online sessions (applies only to the intervention group) and completed the T2 survey (by 28 days after release of T2 at the latest, i.e. 4 weeks after T2).

## 3.3 Methods of analysis

The analyses of the primary and secondary endpoints are performed by means of an analysis of covariance on the differences of the respective scores between time T0 and T2.

## 3.4 Covariates

In addition to the value of the score at T0 (baseline), the following variables are included in the model as covariates: Type of Internet addiction, age, gender and presence of current psychiatric comorbidities (MINI without ADHD).

## 3.5 Missing Data

Missing data at time T2 are replaced with intermediate values (from T1). If the measured value at T1 is also not available, multiple imputation is carried out using regression models. For this purpose, a linear regression model is fitted on the basis of the variables taken into account in the randomisation (AICA-S at baseline (T0), age, gender, type of internet addiction), which makes predictions for the score at time T2. The missing data are replaced by the corresponding predictions. The type of internet addiction is grouped into (1) online streaming services, (2) online gaming, (3) pornography/cybersex, (4) social media and online communities, and (5) all other genres (shopping, information research, gambling and writing emails). The regression weights are unbiased under missing-at-random (MAR) if the factors influencing missingness are part of the regression model. On the other hand, the variability of the imputed data is systematically underestimated. The extent of the underestimation depends on the variance explained and the proportion of missing cases (Little and Rubin, 2002, p. 64). Since it can be assumed that there are further factors that have an influence on the missing data, various sensitivity analyses (see 5.3 Sensitivity analyses) with different replacement strategies, in particular also conservative replacements, are to be calculated in order to estimate the influence on the results.

# 4 Summary of the study data

All continuous variables are summarised by treatment group using the following descriptive statistics: N (non-missing sample size), mean ± standard deviation (SD) (approximately normally distributed variable values) or median, maximum and minimum (if non-normally distributed variable values). The absolute frequencies and percentages (based on the non-missing sample size) of the observed values are reported for all categorical variables.

## 4.1 Description of the patient flow

Figure 2: CONSORT Diagram.

## 4.2 Demographic and baseline variables

The following patient characteristics and baseline covariates before treatment are presented descriptively.

|  | **Randomisation group** | |
| --- | --- | --- |
|  | **IG (n=XX)** | **WCG (n=YY)** |
| **Socio-demographics** | | |
| **Age (in years)** |  |  |
| **Gender**  **-** female  - male  - diverse |  |  |
| **Marital status** |  |  |
| - unmarried without partner |  |  |
| - unmarried with partner |  |  |
| - married |  |  |
| - divorced |  |  |
| - widowed |  |  |
| **Professional situation** |  |  |
| - Full-time employed |  |  |
| - Part-time employed |  |  |
| - Self-employed |  |  |
| - Unemployed |  |  |
| - In vocational training |  |  |
| - Studying at university |  |  |
| - Other (e.g. pupil, pensioner, unable to work) |  |  |
| **Educational level** |  |  |
| - high (≥ 12 years) |  |  |
| - moderate (10 years) |  |  |
| - low (≤ 9 years) |  |  |
| - Currently still in education |  |  |
| **Morbidity-related characteristics** | | |
| **Ever received treatment for a mental or psychiatric disorder** |  |  |
| **Comorbidities (current)** |  |  |
| - Affective disorders (e.g. depression, bipolar disorder) |  |  |
| -Neurotic and stress disorders (e.g. panic disorder, social phobia, obsessive-compulsive disorder),  - Mental and behavioural disorders caused by  psychotropic substances (e.g. alcohol, drug abuse)  -Eating disorders (e.g. anorexia-bulimia nervosa, binge eating) |  |  |
| **Comorbidities (in the past)** |  |  |
| - Affective disorders (e.g. depression, bipolar disorder) |  |  |
| - Neurotic and stress disorders (e.g. panic disorder, social phobia, obsessive-compulsive disorder) |  |  |
| - Mental and behavioural disorders caused by psychotropic substances (e.g. alcohol, drug abuse) - Eating disorders (e.g. anorexia, bulimia nervosa, binge eating) |  |  |
|  |  |  |
| **At least one comorbidity (current)** |  |  |
| **AICA-S** |  |  |
| **AICA-SKI:IBS Interview** |  |  |
| - No disorder, but subjective distress |  |  |
| - Mild disorder |  |  |
| - moderate disorder |  |  |
| - severe disorder |  |  |
| **Extent and type of addictive behaviour** | | |
| **Weekend internet use hrs/day** |  |  |
| **Internet use weekdays hrs/day** |  |  |
| **Average weekly internet use hrs/week** |  |  |
| **Start of problematic internet use (number in years)** |  |  |
| **Internet applications (most problematic)** |  |  |
| - Online games |  |  |
| - Online pornography |  |  |
| - Online streaming |  |  |
| - Social networking sites & chatting |  |  |
| - Other (e.g. shopping, gambling, information search) |  |  |
| **BFI-10** |  |  |
| - Negative emotionality (formerly: neuroticism) |  |  |
| - Extraversion |  |  |
| - Openness |  |  |
| - Agreeableness |  |  |
| - Conscientiousness |  |  |
| **Positive ADHD Screening V1.1** |  |  |

# 5 Efficacy analyses

The analysis is conducted with R (version 4.2.1). All null hypotheses are tested two-sided at the 5% significance level, unless otherwise stated.

## 5.1 (Modified) ITT Analyses

The analysis of the primary endpoint will be conducted as an intention-to-treat (ITT) analysis, i.e. all randomised participants will be included in the analysis, regardless of OMPRIS programme completion or outcome measurement. Missing data will be replaced by imputation with intermediate values (if available from T1). If the measured value at T1 is also not available, multiple imputation is performed using regression models. Our aim is to investigate the de jure hypothesis and evaluate the effectiveness of the treatment. The aim is to estimate the difference in outcome improvement among all randomised subjects between baseline (T0) and the planned endpoint (T2) that is attributable to the original randomised intervention. Thus, we are trying to measure the de jure estimate of the treatment effect.

The secondary endpoints are evaluated in modified ITT populations. These include all randomised subjects who have the respective secondary endpoint at T0 and at T1 or T2, i.e. if the measured value at T2 is missing, it is replaced by the intermediate value at T1, but there is no multiple imputation as provided for the primary endpoint. The number of subjects considered in each analysis is indicated.

Linear model

The observation of the score at time T2 of the i-th participant (i=1,...,n) is given by

$$y_{i}=\beta_{0}+ \beta_{1}\mathrm{trea}t_{i}+\beta_{2}\mathrm{scoreT}0_{i}+\beta_{3}\mathrm{ar}t_{i}+ \beta_{4}\mathrm{se}x_{i}+ \beta_{5}\mathrm{ag}e_{i}+ \beta_{6}\mathrm{psy}K_{i}+e_{i},$$

where

- $\beta_{0}$ Intercept
- $\beta_{1}$ Intervention effect
- $\beta_{2}$ Effect of the score at baseline (T0)
- $\beta_{3}$ Effect for the different types of internet addiction
- $\beta_{4}$ Effect of gender
- $\beta_{5}$ Effekt of ages
- $\beta_{6}$ Effect for the presence of current psychiatric comorbidities

| **Variable** | **Definition** | **Characteristics in the data set** | **Grouping in the model** |
| --- | --- | --- | --- |
| **y** | Score (primary and secondary) measured after intervention (T2) | numeric |  |
| **treat** | Belonging to the intervention group (IG) or waiting control group (WCG) | factorial |  |
| **scoreT0** | Score (primary and secondary) at randomisation (T0) | numeric |  |
| **art** | Type of internet addiction | 1 = Online-Gaming  2 = Online Shopping  3 = Chatting/forums  4 = Emails  5 = Online sex offers  6 = Online-Gambling  7= Online-Communities  8 = Information research  9 = Online-Streaming | 1 = Online-Streaming  2 = Online-Gaming  3 = Online sex offers (e.g. pornographic pictures)  4 = Online communities & chatting/exchanging in forums  5 = Other genres (shopping; writing emails; online gambling; information research) |
| **sex** | Gender of the participant | - male (m)  - female (f)  - diverse (d) | Merging of "f" and "d" due to insufficient group occupation of "d” |
| **age** | Age of the participant at the time of randomisation (T0) | numeric |  |
| **psyK** | Presence of at least one current psychiatric comorbidity:   - MINI_A1_T0 (Major Depression Disorder, current) - MINI_A3_T0 (Major Depression Disorder, recurred) - MINI_C1_T0 (Bipolar-I-disorder, current) - MINI_C3_T0 (Bipolar-II-disorder, current) - MINI_C5_T0 (other bipolar disorder, current) - MINI_D_T0 (panic disorder, current) - MINI_E_T0 (Agoraphobia, current) - MINI_F1_T0 (Social phobia, current, generalised) - MINI_F2_T0 (Social Phobia, current, not generalised) - MINI_G_T0 (obsessive compulsive disorder, current) - MINI_H_T0 (PTSD, current) - MINI_L_T0 (Anorexia nervosa, current) - MINI_M1_T0 (Bulimia nervosa, current) - MINI_M2_T0 (Anorexia nervosa, bulimic type, current) - MINI_N_T0 (Generalised anxiety disorder, current) - MINI_P_T0 (antisocial personality disorder) - BES_T0 (Suspected Binge Eating Disorder) | binary:  - 1= Yes  - 0 = No |  |

## 5.2. Per-Protocol Analysis

All analyses are repeated using the PP analysis set.

## 5.3 Sensitivity analyses

Since it can be assumed that missing data are not "missing-at-random", different sensitivity analyses with different replacement strategies, in particular also conservative replacements, are to be calculated in order to estimate the influence on the results. 
1) Patients for whom the measured value at T1 and T2 are missing will not be considered in the sensitivity analysis.

2) If the measured value at T1 and T2 is missing, T2 is set to the score value at T0.

## 5.4 Subgroup analyses

Instead of subgroup analyses, various moderator analyses are planned, see following section.

## 5.5 Moderator analyses

By means of a moderator analysis, it will be examined whether there are possible predictors (e.g. person-related characteristics) for successful participation in OMPRIS. For this purpose, models with interactions between the intervention and the severity (AICA-S), age, gender and type of addiction are calculated.

1. Dieris-Hirche J, Bottel L, Pape M, *et al*. Effects of an online-based motivational intervention to reduce problematic internet use and promote treatment motivation in internet gaming disorder and internet use disorder (OMPRIS): study protocol for a randomised controlled trial. *BMJ Open* 2021;11:e045840. doi:10.1136/bmjopen-2020-045840 [↑](#footnote-ref-1)
2. Bundesamt für Statistik. Statistisches Jahrbuch Deutschland und Internationales 2019, 2019. Available: https://www.statistischebibliothek. de/ mir/ receive/ DEAusgabe_ mods_ 00004527 [↑](#footnote-ref-2)
3. DAK-Gesundheit. Mediensucht 2020 – gaming und social media in Zeiten von corona. DAK-Längsschnittstudie: Befragung von Kindern, Jugendlichen (12 – 17 Jahre) und deren Eltern. Hamburg,n2020. Available: https://www.dak.de/dak/bundesthemen/computerspielsucht- 2296282. html#/ [↑](#footnote-ref-3)
4. Ko C-H, Yen J-Y. Impact of COVID-19 on gaming disorder: monitoring and prevention. *J Behav Addict* 2020;9:187–9. [↑](#footnote-ref-4)
5. Balhara YPS, Kattula D, Singh S, *et al*. Impact of lockdown following COVID-19 on the gaming behavior of college students. *Indian J Public Health* 2020;64:S172–6. [↑](#footnote-ref-5)
6. Montag C, Wegmann E, Sariyska R, *et al*. How to overcome taxonomical problems in the study of Internet use disorders and what to do with "smartphone addiction"? *J Behav Addict* 2019:1–7. [↑](#footnote-ref-6)
7. Borm GF, Hoogendoorn EH, den Heijer M, *et al*. Sequential balancing: a simple method for treatment allocation in clinical trials. *Contemp Clin Trials* 2005;26:637–45. [↑](#footnote-ref-7)
8. te Wildt B. Entwicklung und evaluation eines Online-Ambulanz- Service Zur Diagnostik und Beratung. Abschlussbericht an das Bundesministerium für Gesundheit, 2018. Available: https:// www. bund esge sund heit smin isterium. de/ fileadmin/ Dateien/ 5_Publikationen/ Drogen_ und_ Sucht/ Berichte/ Abschlussbericht/Abschlussbericht_ OASIS. pdf [↑](#footnote-ref-8)
9. Bottel L, Bielefeld M, Steinbuechel T. Evaluation of an online ambulatory service for Internet addicts (OASIS). *J Behav Addict* 2018;7:45. [↑](#footnote-ref-9)
10. Wölfling K, Müller KW, Beutel M. Reliabilität und validität der skala zum computerspielverhalten (CSV-S). *Psychother Psych Med* 2011;61:216–24. [↑](#footnote-ref-10)
11. Wölfling K, Beutel ME, Müller KW. OSV-S - skala zum onlinesuchtverhalten. In: Geue K, Strauß B, Brähler E, eds. *Diagnostische verfahren in Der psychotherapie*. Göttingen: Hogrefe, 2016: 362–6. [↑](#footnote-ref-11)
12. Wölfling K, Müller KW, Dreier M, *et al*. Efficacy of short-term treatment of Internet and computer game addiction: a randomized

    clinical trial. *JAMA Psychiatry* 2019;76:1018–25. [↑](#footnote-ref-12)
