# Supplementary material for: Efficacy of a short-term webcam-based telemedicine treatment of internet use disorders (OMPRIS): a multicentre, prospective, single-blind, randomised, clinical trial
Source: eClinicalMedicine. 2023 Sep 14;64:102216. doi: 10.1016/j.eclinm.2023.102216 (PMC10514435; doi:10.1016/j.eclinm.2023.102216)
Supplement: OMPRIS Study group [file mmc4.docx]

**Further members of the OMPRIS Study group**

**First names Surnames**

Annika Best

Raffaela Böswald

Lorraine Cornelsen

Michael Dreier

Sofie Groen

Alicia Hemmersbach

Vivienne Hillerich

Ina Krahn

Dennis Lowin

Alicia Menze

Silke Neusser

Nehle Penning

Heribert Sattel

Christian Suelmann

Marianne Tokic

Julia Weretecki

The further members of the OMPRIS research group should be listed in PubMed.
